# Incidence of lost to follow up among HIV-positive children on antiretroviral therapy in Ethiopia: Systematic review and meta-analysis

**DOI:** 10.1371/journal.pone.0304239

**Published:** 2024-05-22

**Authors:** Desalegn Girma, Zinie Abita, Lidya Gutema Lemu, Daniel Asmelash, Getachew Mesfin Bambo, Melesew Setegn Alie, Gossa Fetene Abebe

**Affiliations:** 1 Department of Midwifery, College of Health Science, Mizan-Tepi University, Mizan-Teferi, Ethiopia; 2 Department of Public Health, College of Health Science, Mizan-Tepi University, Mizan-Teferi, Ethiopia; 3 Department of Medical Laboratory, College of Health Science, Mizan Tepi University, Mizan-Teferi, Ethiopia; Management Sciences for Health (MSH), ETHIOPIA

## Abstract

**Background:**

At the end of 2022, globally, only 46% of children (aged 0–14 years) on ART had suppressed viral loads. Viral load suppression is crucial to reduce HIV-related deaths. To suppress the viral load at the expected level, children must be retained in ART treatment. Nevertheless, lost to follow-up from ART treatment continues to be a global challenge, particularly, in developing countries. Previously, primary studies were conducted in Ethiopia to assess the incidence of lost to follow-up among HIV-positive children on ART treatment. However, variations have been seen among the studies. Therefore, this systematic review and meta-analysis aimed to estimate the pooled incidence of lost to follow-up among HIV-positive children on ART and identify its associated factors in Ethiopia.

**Methods:**

We searched PubMed, HINARI, Science Direct, Google Scholar, and African Journals Online to obtain articles published up to November 20, 2023. Critical appraisal was done using the Joanna Briggs Institute checklist. Heterogeneity was identified using I-square statistics. Funnel plot and Egger’s tests were used to identify publication bias. Data was presented using forest plots and tables. Random and fixed-effect models were used to compute the pooled estimate.

**Results:**

Twenty-four studies were included in the final analysis. The pooled incidence of lost to follow-up among HIV-positive children on ART was 2.79 (95% CI: 1.99, 3.91) per 100-child-year observations. Advanced HIV disease (HR: 2.20, 95% CI: 1.71, 2.73), having opportunistic infection (HR: 2.59, 95% CI: 1.39; 4.78), fair or poor ART treatment adherence (HR: 2.92, 95% CI: 1.31; 6.54) and children aged between 1–5 years (HR: 2.1,95% CI: 1.44; 2.95) were factors associated with lost to follow up among HIV positive children on ART.

**Conclusions:**

The overall pooled incidence of lost to follow-up among HIV-positive children on ART is low in Ethiopia. Therefore, counseling on ART drug adherence should be strengthened. Moreover, emphasis has to be given to children with advanced HIV stage and opportunistic infection to reduce the rate of lost to follow up among HIV-positive children on ART.

**Trial registration:**

Registered in PROSPERO with ID: CRD42024501071.

## Introduction

Human Immunodeficiency Virus (HIV) continues to be one of the global public health concerns, particularly in sub-Saharan Africa. At the end of 2022, an estimated 1.5 million children aged 0–14 years were living with HIV infection and about 130,000 of them were newly infected [1]. In the same year, globally, about 84,000 children died from HIV-related diseases [2]. Despite multiple efforts made, about 43% of children living with HIV were not receiving treatment [3].

To avert the burden of HIV, different efforts have been made globally. In 2015, the United Nations, under its sustainable development goal (SDGS), set a global target to end HIV epidemic by 2030 through the provision of antiretroviral therapy (ART) [4]. ART has profound importance in prolonging the life of people living with HIV infection, mainly, by suppressing the viral load, improving the immune system, and reducing the risk of opportunistic infections [5]. To increase the accessibility and coverage of ART, currently, the United Nations has commenced a 95-95-95 ambitious treatment target for the year 2025, which implies that 95% of people living with HIV know their status, 95% of people living with HIV who know their status are receiving treatment and 95% of people on treatment have suppressed viral loads by the year 2025 [6].

To achieve the aforementioned ambitious treatment targets, HIV-infected children must be retained in a cohort of ART treatment and have regular follow-ups [7]. During follow-up, children are checked for clinical progression, ART side effects, drug adherence, and viral load suppression and should be counseled for optimal drug utilization [8].

Lost to follow-up (LTFU) from ART is one of the global public health concerns, particularly in developing countries. A systematic review conducted in resource-limited settings found that about 5–29% of children living with HIV were lost from ART within the first 12 months of ART initiation [9]. In Sub-Saharan African countries, the proportion of LTFU among HIV-positive children after two years of ART initiation varied from the lowest 9.0% in Southern Africa to the highest 21.8% in West Africa [10]. Children who drop out of the treatment are at an increased risk of drug-resistant virus and treatment failure. These can jeopardize the effectiveness of HIV treatments and increase HIV-related deaths [11, 12].

In Ethiopia, there is a paucity of evidence regarding the national incidence of LTFU among HIV-positive children on ART. Moreover, previous primary studies also confirmed that the incidence of LTFU among HIV-positive children on ART varied across regions in Ethiopia [13–21], ranging from 3.3 per 100 child years in the Oromia region [21] to 6.3 per 100 child years in the Amhara region [15]. Conducting an aggregated study using those primary studies is important to know about the national burden of LTFU among HIV-positive children on ART. Therefore, the main objective of this systematic review and meta-analysis is to estimate the pooled incidence of LTFU among HIV-positive children on ART and identify its associated factors in Ethiopia.

## Methods

### Search strategy

The result was reported using the Preferred Reporting Items for Systematic Review and Meta-Analysis (PRISMA) guideline [22]. We searched PubMed, HINARI, Science Direct, Google Scholar, and African Journals Online to obtain relevant studies. Online database searching was done on November 20, 2023. The following terms and phrases search as “incidence rate”, “lost to follow-up”, “LTFU”, “treatment outcome”, “attrition”, “pediatrics”, “children” “child”, “**newborn”,** “Human Immunodeficiency virus”, “HIV”, “ART”, “antiretroviral therapy” “antiretroviral drugs”, “AIDS drugs”, “Anti-HIV drugs” and “Ethiopia” were the main key search terms used in this systematic review and meta-analysis. We used Boolean operators such as “AND” and “OR” during database searching **(S1 File)**.

### Eligibility criteria

The inclusion criteria were: 1) studies conducted in Ethiopia, 2) studies that report the incidence of LTFU or the number of LTFU for children aged 0–14 years living with HIV, 3) studies that report the child person year, 4) studies published in English languages, 5) studies that report at least one predictors with hazard function and 6) studies available at the electronic source up to November 20, 2023 were included in the study. On the other hand, studies that didn’t report the child person’s years or studies that report predictors other than hazard function were excluded from the study. Furthermore, citations without abstract and/or full-text, anonymous reports, editorials, and qualitative studies were excluded from the analysis.

### Data extraction

All studies identified via the online database were exported to EndnoteX7 to identify and remove duplication. A standardized data extraction tool was used and independently extracted by four authors (GF, ZA, GM, and MS). Any disagreements among the data extractors were discussed and it was handled by the principal authors (DG). From each study, the author’s name, publication year, the event or number of LTFU, study region, study design, the total person year, incidence rate, and the predictor of LTFU with hazard ratios were extracted.

### Quality assessment/Critical appraisal

Quality appraisal was done using the Joanna Briggs Institute (JBI) Critical Appraisal Checklist for cohort study design [23]. The qualities of the primary studies were independently assessed by two authors (LG and DA). Any discrepancy between the two authors was handled by taking the mean score of the two authors. The tool has Yes, No, Unclear, and Not Applicable options: “1” is given for “Yes” and “0” is given for other options. The scores were summed and changed to percentages. Studies with >50% quality scores were included in this meta-analysis. Finally, twenty-four studies that received a quality score of >50% were included in the final analysis **(S2 File)**.

### Outcome measurement

The first outcome was the incidence of LTFU among HIV-positive children on ART. The incidence of LTFU among HIV-positive children on ART was calculated by dividing the number of children who lost from ART treatment for one to three months by the total child follow-up years and multiplying it by 100. Identifying the associated factors of LTFU among HIV-positive children on ART was the second outcome of this study. Accordingly, the hazard ratio of predictors with its 95% confidence intervals (CI) was extracted from the original studies to compute the pooled hazard ratio of predictors.

#### Lost to follow-up

When HIV-positive children miss an appointment or drug pick-up for one month to three months and are not yet classified as dead or transferred out [24].

#### Follow-up period (time)

Is measured from the beginning of the study until the event (LTFU) occurs, transferred-out, death, and the study ends.

#### Advanced HIV disease

Children older than five years whose WHO clinical stages are III and IV. Whereas, children younger than five years living with HIV are considered as having advanced HIV disease, regardless of the clinical stages. **Mild WHO clinical stages**: HIV-positive children whose WHO clinical stages are stages I and II [24].

**ART adherence. Good (> 95%)—**if missed doses is ≤ 2 doses of 30 doses or ≤ 3 doses of 60 doses; **Fair: (85–94%)** if missing doses is between 3–4 of 30 doses or 4–9 of 60 doses; **poor: (< 85%)** if missed doses are >5 doses of 30 doses or 10 and above doses of 60 doses of ART drug [24].

### Statistical analysis

Data entry was done using Microsoft Excel 2013 and then imported into R software version 4.1.3 for further analysis. Meta-package was used to analyze the data. I-square was used to check heterogeneity between studies [25]. Heterogeneity was declared as low, medium, and high if the I^2^ value was 25%, 50%, and 75%, respectively [26]. Subgroup analysis was done using the study region with evidence of heterogeneity. Univariate meta-regression analysis was done using publication years and sample size to identify the possible source of heterogeneity. Sensitivity analyses were done by omitting individual studies to detect the contribution of each study in the final pooled incidence of LTFU among HIV-positive children on ART. Funnel plot visual inspection was done to identify publication bias. Finally, the Egger test was done to assess any significant publication bias. Further, Trim and fill analyses were conducted to correct publication bias. Tables and forest plots were used to present data. The random and fixed effect models were used to compute the pooled estimates. Results are presented using the random effect model (if there is heterogeneity among studies) and fixed-effect (if there is no heterogeneity among studies).

## Results

### Characteristics of included studies

**Fig 1:**
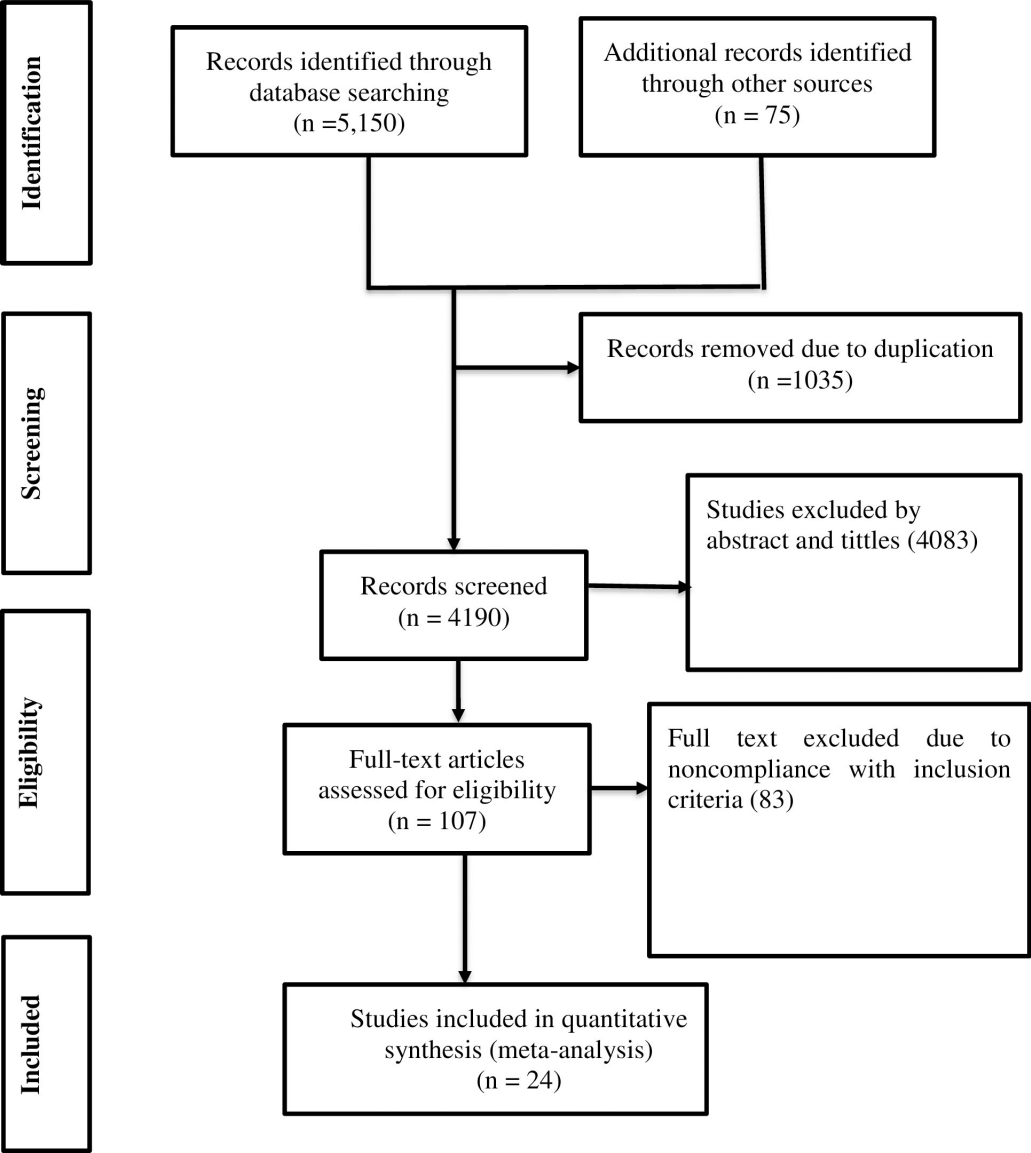
PRISMA flow chart describing screening protocols of studies for meta-analysis.

A total of 5,225 articles were identified from PubMed, HINARI, Science Direct, Google Scholar, and African Journals Online. Of these, 2,514 studies were from PubMed, 1,127 studies were from HINARI, 1,509 studies were from Science Direct, and the rest 75 studies were identified from Google Scholar and African Journals online. From these studies, 1035 studies were excluded due to duplication. From the remaining 4190 articles, 4083 studies were excluded as not being relevant to the study after reviewing the titles and abstracts. The rest 107 articles were assessed by reviewing the full text. Finally, twenty-four studies were eligible and incorporated in the final analysis (**Fig 1**) [13–21, 27–41]. All studies were conducted using the cohort study design. These studies were done from different parts of Ethiopia (Addis Ababa, Amhara, Oromia, SNNPR (South Nation, Nationalities and People Region), and, Tigray) **(Table 1)**.

**Table 1:** Characteristics of studies included in the meta-analysis for the pooled incidence of LTFU among HIV-positive children on ART, Ethiopia, 2023.

Author	Region	Sample sizes	Number of LTFU	Follow-up time in month (IQR)	PMO	PYO	IR per 100 child years
Mulgeta et al (2017) [27]	Addis Ababa	757	92	62.0–83.0	49344	4112	
Edessa et al (2015) [28]	Oromia	305	22	18–30	7, 312	609	---
Adem et al (2014) [29]	Oromia	560	46	29–62	24936	2078	---
Bimer et al (2021) [13]	SNNPR	254	70	1–84	8145.33	678.78	---
Chanie et al (2022) [14]	Amhara	344	76	(4–167	19,081	1590.1	4.8
Menshw et al (2021) [15]	Amhara	488	101	-------	-------	---	6.3
Fetene et al(2018) [16]	Amhara	533	46	42–11	15288	1274	3.6
Sidamo et al (2017) [30]	SNNPR	421	43	24–80	21,175	1764.58	---
Haile(2021) [31]	SNNPR	429	38	32–122	30595.2	2549.6	---
Fisiha et al (2020) [17]	Amhara	361	79	14–70	15369.6	1280.8	6.2
Sifr et al (2021) [18]	SNNPR	143	18	-------	-------	356.06	5
Bankere et al (2022) [21]	Oromia	269	43	24–92	15588	1299	3.3
Alebel et al (2020) [32]	Amhara	538	38		14,600	1216	---
Hibstie et al (2020) [19]	Amhara	408	70	2–136	18,755	1562.9	4.5
Melaku et al (2017) [33]	Amhara	6815	2090	------	------	---	---
Koye et al (2012) [34]	Amhara	549	32	1–62	12300	1025	---
Gebremedihn et al (2013) [35]	Tigray	416	23	17–50	14,235	1186.25	---
Gemech et al (2022) [36]	SNNPR	284	32	1–120	15086.04	1257.17	---
Tagesse et al (2020) [37]	Addis Ababa	410	20	18–44	13236	1103	---
Biru et al (2018) [20]	Adiss Ababa	304	18	10–13	3452.4	287.7	9.12
Atallel et al (2018) [38]	Amhara	271	25	1–144	14012.04	1167.67	---
Biyazin et al (2022) [39]	Amhara	251	16	60	7512	626	---
seid (2023) [40]	SNNPR	261	11	43–107	18,955	9477.5	---
Alemu et al(2022) [41]	Amhara	415	18	3–48	8700.5	725.04	---

LTFU: Lost to follow up, IQR: Inter Quartile Range, PMO: Person Month Observation, PYO: Person Year Observation, IR: incidence rate

### The pooled incidence of LTFU among HIV‑positive children on ART

**Fig 2:**
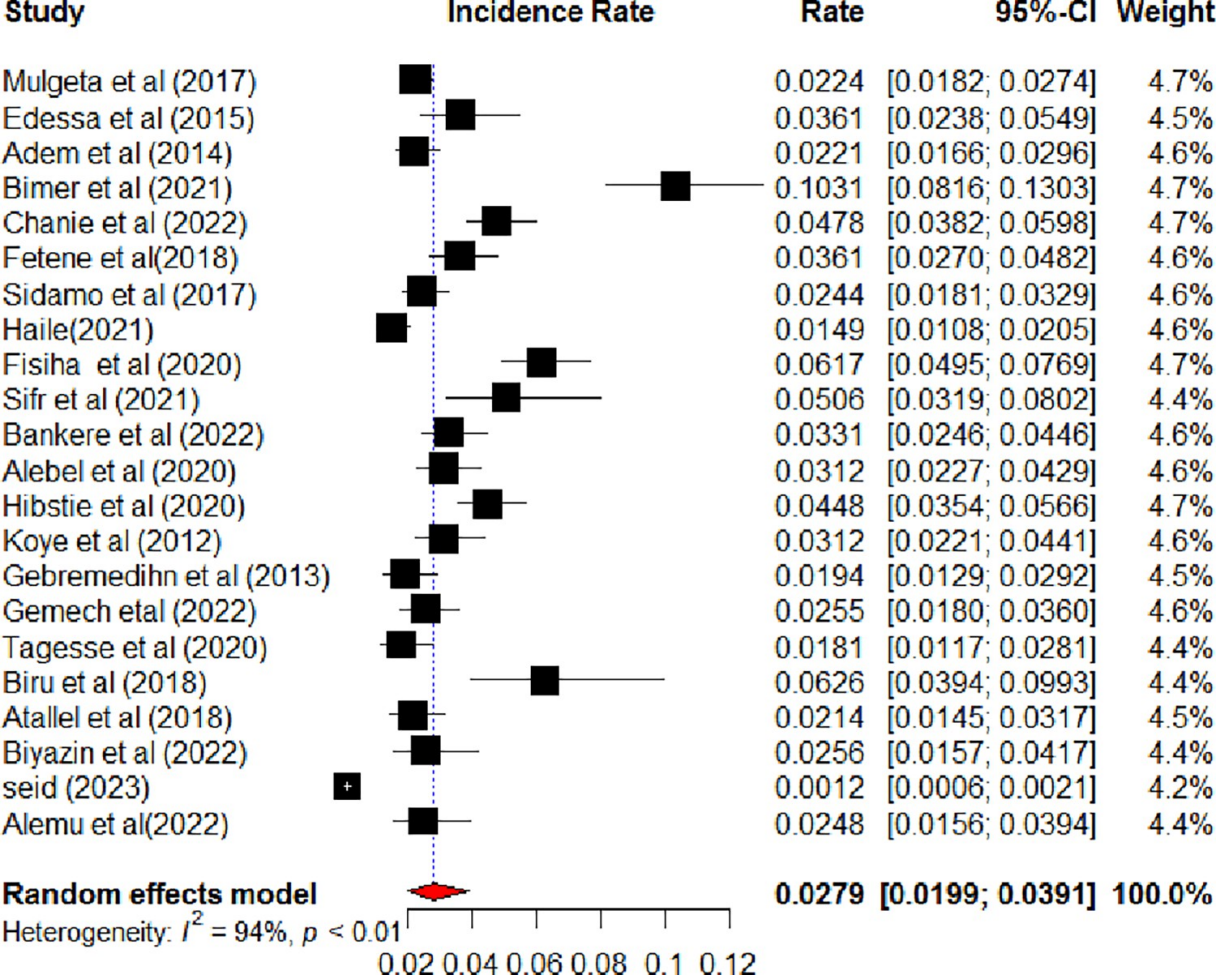
The forest plots show the incidence of lost follow-up among HIV-positive children on ART, Ethiopia, 2023.

**Fig 3:**
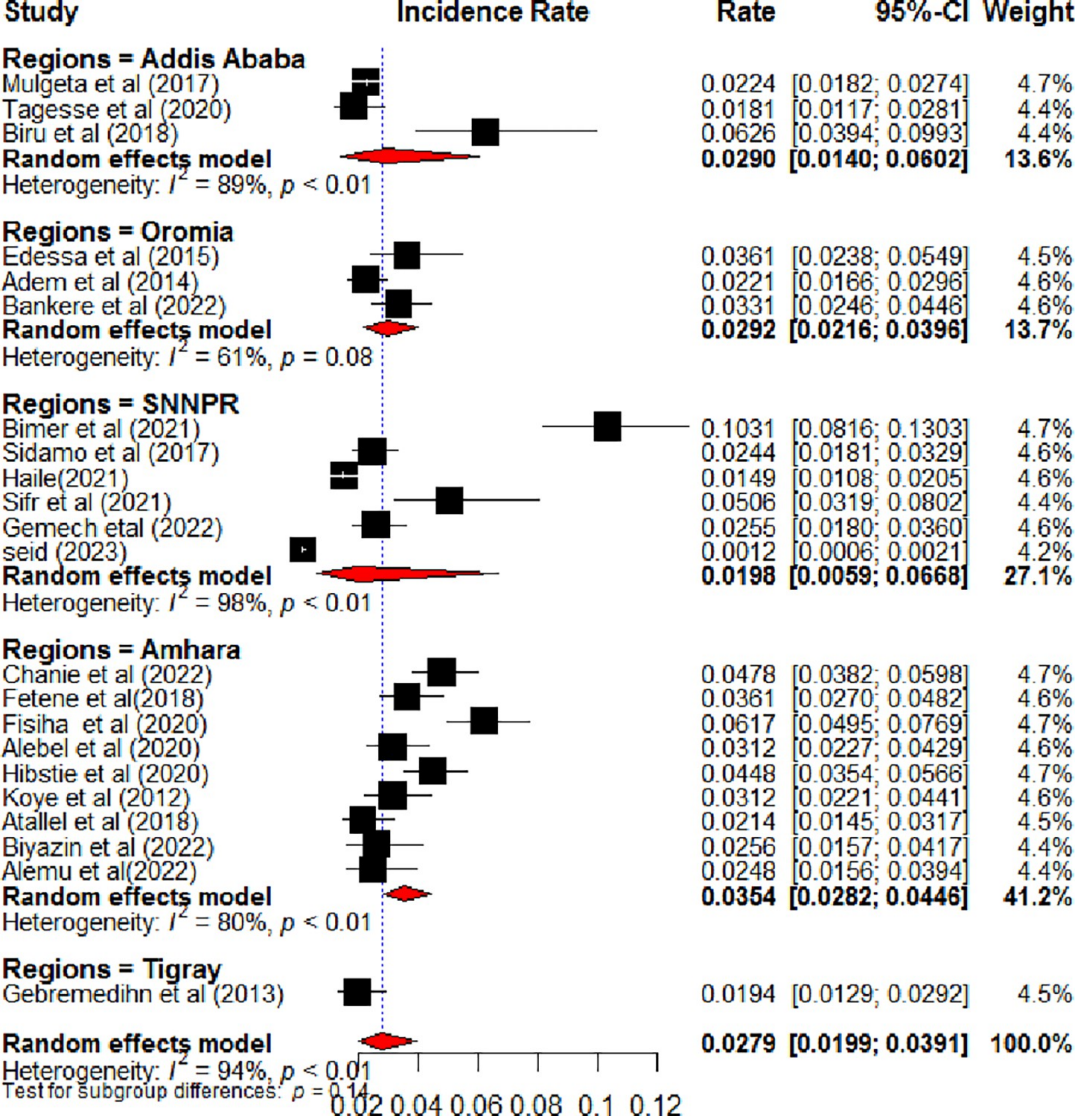
Forest plot shows the subgroup analysis of the incidence of lost to follow-up among HIV-positive children on ART by study regions, Ethiopia, 2023.

**Fig 4:**
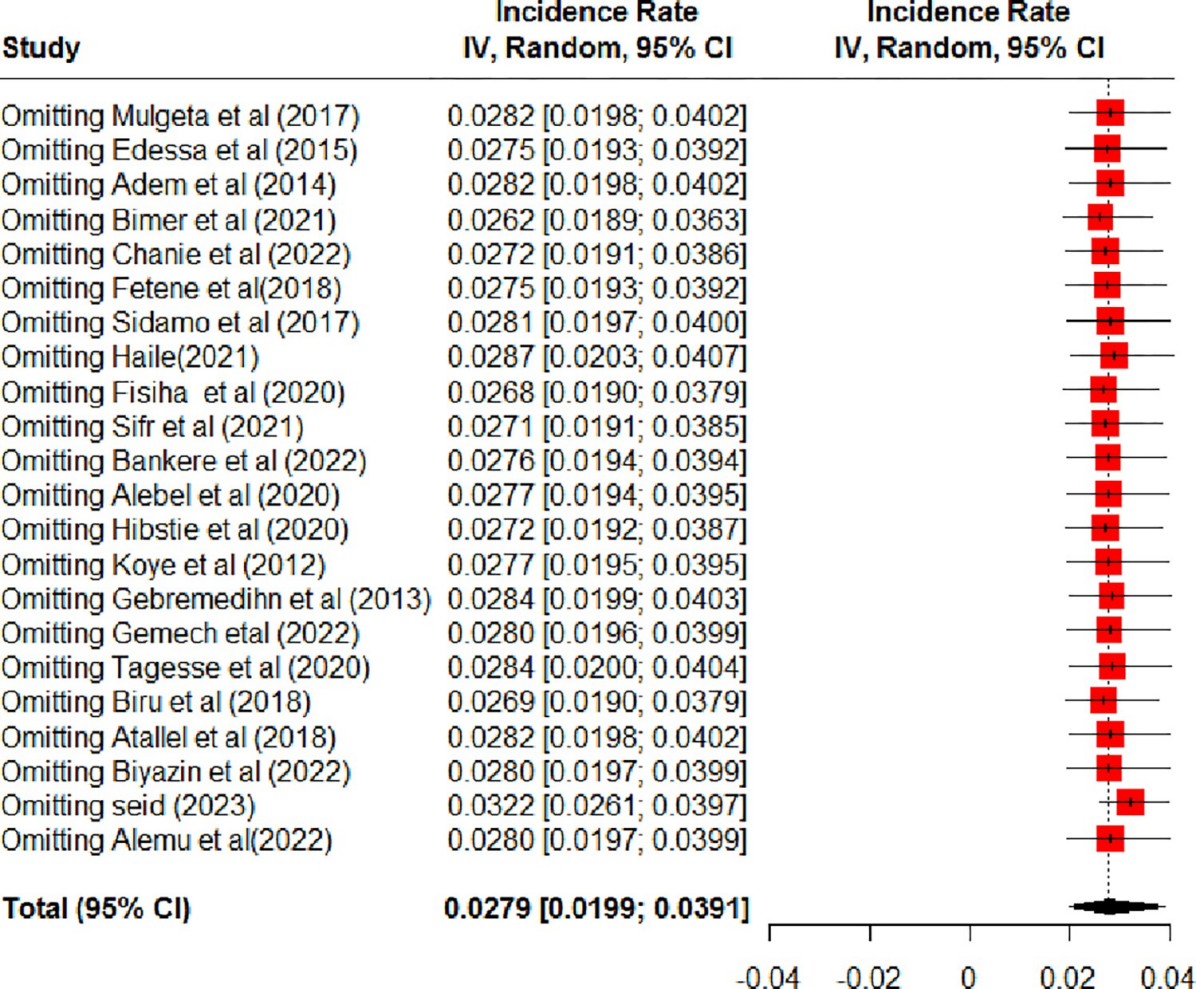
Sensitivity analysis for the incidence of lost to follow-up among HIV-positive children on ART, Ethiopia, 2023.

Twenty-two studies were included to estimate the pooled incidence of LTFU among HIV-positive children on ART in Ethiopia [13, 14, 16–21, 27–32, 34–41]. Accordingly, the pooled incidence of LTFU among HIV-positive children on ART in Ethiopia was 2.79 (95% CI: 1.99, 3.91) per 100-child-year observations using a random effect model. Heterogeneity (I^2^ = 94%, P-value <0.01) was identified **(Fig 2)**. Hence, subgroup analysis was done based on the study regions. Accordingly, the incidence of LTFU among HIV-positive children on ART ranged from 1.98 per 100 child years in SNNPR to 3.54 per 100 child years in the Amhara region **(Fig 3)**. Univariate meta-regression analysis was done to identify the possible source heterogeneity using the publication years and sample size. Of these factors, none of them were statistically significant **(Table 2)**. Finally, sensitivity analysis was done. In sensitivity analysis, except for one study [40], nearly all studies have equal contributions to the pooled incidence of LTFU among HIV-positive children on ART in Ethiopia **(Fig 4)**.

**Table 2:** Meta-regression analysis using publication year and sample size for the possible source of heterogeneity of LTFU among HIV-positive children on ART, Ethiopia, 2023.

Variables	Coefficients	P-value
Publication years	-0.0405 (-0.1613, 0.0519)	0.3
Sample size	0.0010 (- 0.0006,0.0026)	0.23

### Publication bias

**Fig 5:**
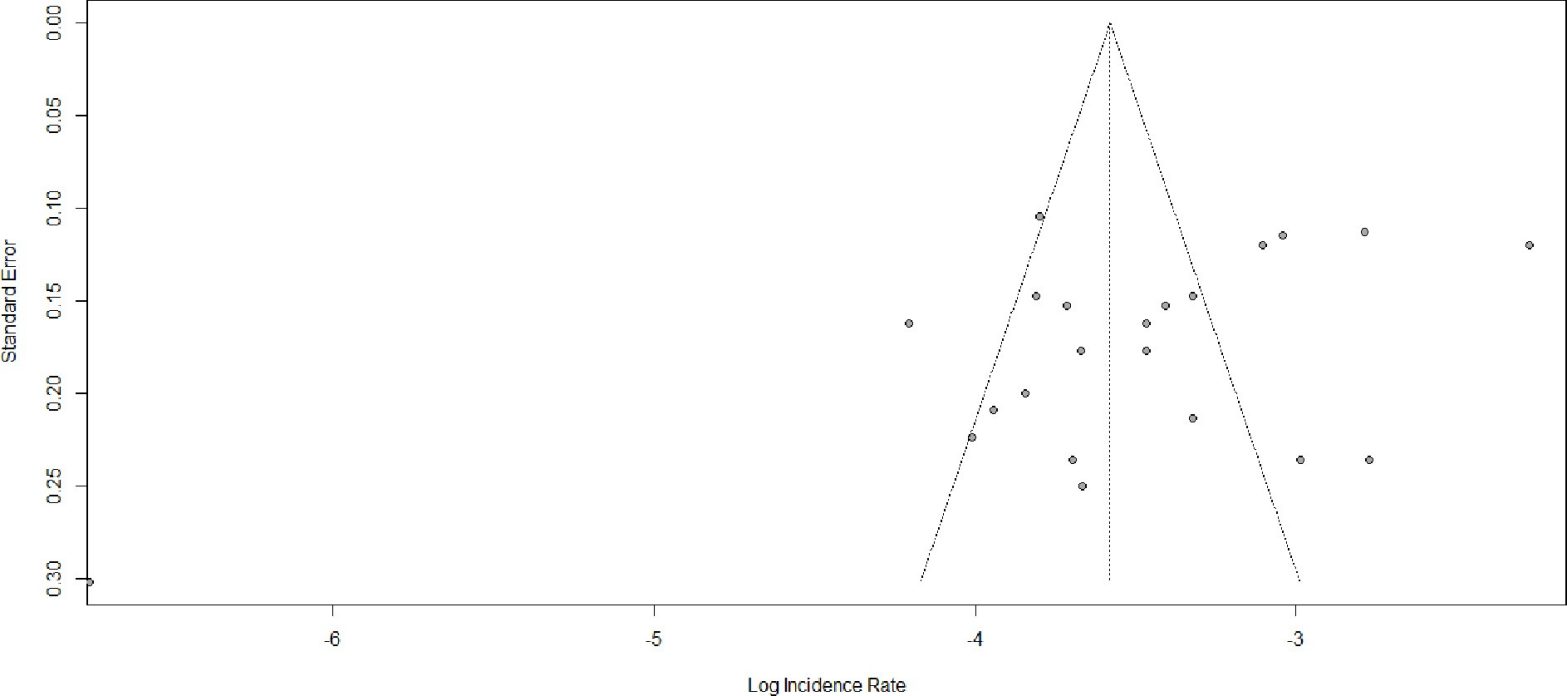
Funnel plot showing publication bias among studies used to compute the pooled incidence of lost to follow-up among HIV-positive children on ART, Ethiopia, 2023.

**Fig 6:**
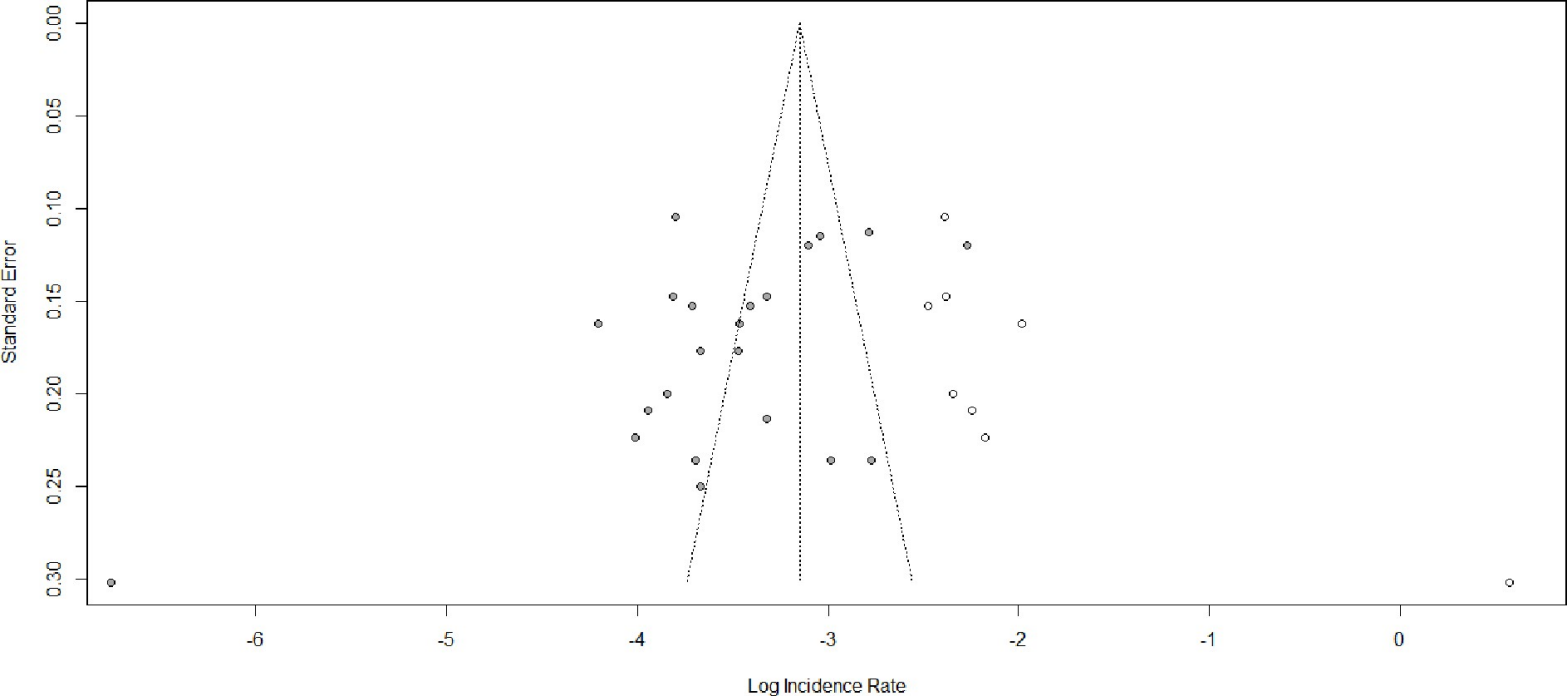
Shows the trim fill analysis for the incidence of lost to follow-up among HIV-positive children on ART, Ethiopia, 2023.

Asymmetric distribution was displayed in the funnel plot visual inspection **(Fig 5)**. The Egger test also shows a statistically significant publication bias with B_0_ = -2.36, p-value = 0.02. Due to the presence of statically significant publication bias, meta-trim and fill analysis were done. Hence, eight studies were filled and the pooled incidence of LTFU among HIV-positive children on ART became 4.29 (95% CI: 2.87; 6.41) per 100 child years (**Fig 6**).

### Factors associated with LTFU among HIV-positive children on ART

In this systematic review and meta-analysis, seven studies were incorporated to identify the factors associated with LTFU among children on ART [15–17, 19–21, 33]. Advanced HIV disease, poor or fair ART treatment adherence, history of opportunistic infection, and age between 1–5 years were factors associated with a higher hazard of LTFU among HIV-positive children on ART. Accordingly, the likelihood of LTFU was 2.20 times (HR: 2.20, 95% CI: 1.71, 2.73) higher among children with advanced HIV disease as compared to children with mild WHO clinica stages [15, 17, 33]. The hazard of LTFU was 2.92 times (HR: 2.92, 95% CI: 1.31; 6.54) higher among children with poor or fair ART treatment adherence as compared to children with good ART treatment adherence [15, 17, 19]. The hazard of LTFU was 2.1 times (HR: 2.1,95% CI:1.44; 2.95) higher among children aged between 1–5 years as compared to children aged ≥ 5 years [15, 16, 20]. The likelihood of LTFU was 2.59 times (HR: 2.59, 95% CI:1.39; 4.78) higher among children who had opportunistic infection as compared to their counterparts [16, 21] **(Table 3)**.

**Table 3:** Predictors of lost to follow up among HIV positive children on ART, Ethiopia, 2023.

Predictors	Included studies	HR (95% CI)	Pooled HR (95% CI)		Heterogeneity
Advanced HIV disease	Menshw et al (2021)	2.2 0 (1.40; 3.34)	2.20 (1.71,2.73)	I^2^ = 0%, p-value = 0.94	
	Fisiha et al (2020)	2.00 (1.10; 3.10)			
	Melaku et al (2017)	2.20 (1.6; 3.10)			
Poor or fair ART treatment adherence	Menshw et al (2021)	6.6 0 (4.11; 10.56)	2.92 (1.31; 6.54)	I^2^ = 90.8%, p-value < 0.001	
	Fisiha et al (2020)	1.70 (1.10; 2.11)			
	Hibstie et al (2020)	2.30 (1.40; 3.70)			
Having Opportunistic infection	Fetene et al(2018)	2.26(1.08; 4.71)	2.59 (1.39; 4.78)	I^2^ = 0%, p-value = 0.51	
	Bankere et al (2022)	3.54 (1.152; 10.87)			
Age between 1–5 years	Menshw et al (2021)	1.60 (1.05; 2.46)	2.10 (1.44; 2.95)	I^2^ = 57.5%, p-value = 0.095	
	Fetene et al(2018)	3.86 (1.73; 8.61)			
	Biru et al (2018)	3.76 (1.16; 12.27)			
Rural residence	Hibstie et al (2020)	3.20 (2.00; 5.50)	1.15 (0.15; 8.84)	I^2^ = 95%. p-value < 0.001	
	Melaku et al (2017)	0.40 (0.20; 0.90)			
	Biru et al (2018)	3.57 (11.64; 11.64)			

## Discussion

This systematic review and meta-analysis unveiled the pooled incidence of LTFU among HIV-positive children on ART in Ethiopia. Accordingly, the pooled incidence of LTFU among HIV-positive children on ART is found to be 2.79 (95% CI: 1.99, 3.91) per 100-child-year observations. The incidence is lower than the studies conducted in Asia (4.2 per 100 child years) [42], in Uganda (12.6 per 100 child years) [43], in Kenya (14.65 per 100 child years [44], in South Africa (10.8 per 100 person-years) [45], in Malawi (12.6/ per 100 person-years) [46], in Tanzania (18.2 per 100 person-years) [47], in South Africa (5.0 per 100 person-years) [48], in Nigeria (40 per 100 child-years) [49], in western Kenya (18.4 per 100 person-years) [50], in Côte d’Ivoire’s(9.3 per 100 person-years) [51] and in Mozambique (6.9/per 100 person-years) [52]. This can be justified that LTFU was considered when HIV-positive children interrupt ART treatment and with unknown tracing outcomes. Thus, the low incidence of LTFU among HIV-positive children on ART might be due to the improvement in tracing, recording, and reporting systems of health institutions for people living with HIV.

In this systematic review and meta-analysis, the hazard of LTFU is higher among children with advanced HIV disease as compared to children with mild WHO clinical stages. The finding is supported by studies conducted elsewhere [49, 53–55]. This can be justified as children with advanced HIV stage are at higher risk of developing opportunistic infections that cause unregistered HIV-related morbidity and mortality.

In this systematic review and meta-analysis, children with poor or fair ART treatment adherence have a higher hazard of LTFU than children with good ART treatment adherence. The finding is consistent with studies conducted elsewhere [56–58]. This is the fact that ART can suppress viral replication, boost immune function, and prevent opportunistic infection [59, 60]. Such that, fair or poor ART treatment adherence can open a window for viral replication, cause HIV viral resistance, decrease drug effectiveness, and cause treatment failure. This increases the risk of opportunistic infection and unregistered deaths.

The hazard of LTFU is higher among children who develop opportunistic infections as compared to their counterparts. The fact that opportunistic infections occur in advanced HIV disease, which further exacerbates the clinical outcome of people living with HIV [61]. Thus, the poor improvement in children with opportunistic infection may make parents feel hopeless or careless and they may fail to bring their child for treatment follow-up. This implies that strict and frequent follow-ups are needed for children with opportunistic infection than children without opportunistic infection.

In this systematic review and meta-analysis, children aged between 1–5 years are at an increased risk of LTFU from ART treatment as compared to children aged ≥ 5 years. The finding is supported by studies conducted elsewhere [55, 62, 63]. This might be due to the immature immune system in infants and young children increases the risk of rapid progression of HIV disease than older children. Thus, infants and young children are highly susceptible to opportunistic infections, which indirectly increases the incidence of LTFU from ART treatment.

The clinical and public health implications of this systematic review and meta-analysis are to take prompt intervention against the identified factors and response to reduce the burden of LTFU among HIV-positive children on ART and increase ART retention, later, to reduce HIV-related deaths. Therefore, researchers, program implementers, and policymakers should consider the aforementioned factors in their strategic plans.

### Limitations

This systematic review and meta-analysis have the following limitations: in this analysis, articles published only in English were included. Only five regions were included in the analysis, such that some regions may not be represented. Moreover, some variables associated with LTFU among HIV-positive children on ART were excluded from the analysis because it reported in only one primary article and/or classified in a different way from the included articles. Furthermore, only seven studies reported the predictors of LTFU among HIV-positive children on ART, such that limited factors were identified. Finally, because of the previous primary studies didn’t report the event (number of LTFU) at 6 months, 12 months, 24 months, and more, the segregated incidence of LTFU among HIV-positive children on ART was not pooled for the respective months.

## Conclusion

The overall pooled incidence of LTFU among HIV-positive children on ART is low in Ethiopia. Advanced HIV disease, having an opportunistic infection, fair or poor ART treatment adherence, and children aged between 1–5 years were factors associated with LTFU among HIV-positive children on ART. Therefore, counseling on ART drug adherence should be strengthened. Moreover, emphasis has to be given to children with advanced HIV stage and opportunistic infection to reduce the rate of LTFU among HIV-positive children on ART.

## Supporting information

S1 Checklist PRISMA 2020 checklist. (DOCX)

S1 File Search terms summary. (DOCX)

S2 File Critical appraisal of studies included in the systematic review and meta-analysis for pooled incidence of lost to follow-up among HIV-positive children on ART, Ethiopia, 2023. (DOCX)

## Abbreviations

ART: Antiretroviral therapy
HIV: Human immunodeficiency virus
AIDS: Acquired Immune Deficiency Syndrome
PYO: Person-year observation
PMO: Person-month observation
WHO: World Health Organization
SNNPR: South Nation, Nationalities and People Region
JBI: The Joanna Briggs Institute Critical Appraisal Checklist
PRISMA: Preferred Reporting Items for Systematic Review and Meta-Analysis Statement
LTFU: lost to follow u
HR: hazard ratio and
CI: confidence interval
