# Supplementary material for: Incidence of lost to follow up among HIV-positive children on antiretroviral therapy in Ethiopia: Systematic review and meta-analysis
Source: PLoS One. 2024 May 22;19(5):e0304239. doi: 10.1371/journal.pone.0304239 (PMC11111029; doi:10.1371/journal.pone.0304239)
Supplement: S1 File — (DOCX) [file pone.0304239.s002.docx]

| **Database** |  | **Query** | **Items found** |
| --- | --- | --- | --- |
| PubMed | **#1** | (((((antiretroviral therapy[MeSH Terms]) OR (ART)) OR (antiretroviral drugs)) OR (AIDS drugs)) OR (Anti-HIV drugs))) OR (Anti-AIDS drugs) | 312,237 |
|  | **#2** | **((((Human Immunodeficiency virus[MeSH Terms]) OR (HIV-infected)) OR (HIV positive)** | 474,18 |
|  | **#3** | **(((loss to follow-up) OR (treatment outcome)) OR (LTFU))) OR (attrition)** | 1,700,053 |
|  | **#4** | **((children) OR (child))) OR (pediatric)** | 3,565,900 |
|  | #5 | Ethiopia | 40,613 |
|  | **#6** | **#1 OR #2 AND #3 AND #4 AND #5** | **2,514** |
| HINARI |  | ((antiretroviral therapy) AND ((loss to follow-up) OR (treatment outcome) OR (attrition)) AND ((children) OR (pediatrics) OR (child)) | **1,127** |
| Science Direct |  | ("HIV infection" OR "antiretroviral therapy") AND ( "loss to follow-up" OR "treatment outcome" OR " attrition") AND ("Children") | **1,509** |
| Google Scholar |  | Incidence of lost follow up AND Children on Antiretroviral Therapy | **65** |
|  |  | attrition rate AND Children on Antiretroviral Therapy |  |
|  |  | Outcome AND Children on Antiretroviral Therapy |  |
|  |  | Predictor or factor of lost to follow up AND Antiretroviral Therapy |  |
| African journal online |  | Incidence of lost follow up AND Children on Antiretroviral Therapy | **10** |
| **Total** | | | **5,225** |

**S1 File**: Search terms summary.
