# Supplementary material for: Incidence of lost to follow up among HIV-positive children on antiretroviral therapy in Ethiopia: Systematic review and meta-analysis
Source: PLoS One. 2024 May 22;19(5):e0304239. doi: 10.1371/journal.pone.0304239 (PMC11111029; doi:10.1371/journal.pone.0304239)
Supplement: S2 File — (DOCX) [file pone.0304239.s003.docx]

**S 2 File**: critical appraisal of studies included in the systematic review and meta-analysis for pooled incidence of lost to follow up among HIV positive children on ART, Ethiopia, 2023.

| Include studies | Eleven JBI Critical Appraisal Checklist for cohort Studies: The tool has Yes, No, Unclear, and Not Applicable options: “1” is given for “Yes” and “0” is given for other options | | | | | | | | | | | | | | | | | | | | | | | | | |
| --- | --- | --- | --- | --- | --- | --- | --- | --- | --- | --- | --- | --- | --- | --- | --- | --- | --- | --- | --- | --- | --- | --- | --- | --- | --- | --- |
|  | Q1 | | | Q2 | | | Q3 | | Q4 | | Q5 | | | Q6 | | Q7 | | Q8 | | Q9 | | Q10 | | Q11 | | Over all |
|  | R1 | R2 | R1 | | R2 | R1 | | R2 | R1 | R2 | R1 | R2 | | R1 | R2 | R1 | R2 | R1 | R2 | R1 | R2 | R1 | R2 | R1 | R2 |  |
| Mulgeta et al (2017) | Y | Y | Y | | Y | Y | | Y | U | U | U | N | Y | | Y | Y | Y | Y | Y | N | Y | N | N | Y | Y | 8/11 (72.72%) |
| Edessa et al (2015) | Y | Y | Y | | Y | Y | | Y | Y | Y | Y | Y | Y | | Y | Y | Y | Y | Y | U | N | U | N | Y | Y | 9/11 (81.82%) |
| Bimer et al (2021) | Y | Y | Y | | Y | Y | | Y | Y | Y | Y | Y | Y | | Y | Y | Y | Y | Y | Y | Y | U | U | Y | Y | 10/11 (90.9%) |
| Chanie et al (2022) | Y | Y | Y | | Y | Y | | Y | N | Y | Y | Y | Y | | Y | Y | Y | Y | Y | U | U | Y | Y | Y | Y | 10/11 (90.9%) |
| Adem et al (2014) | Y | Y | Y | | Y | Y | | Y | U | U | U | N | Y | | Y | Y | Y | Y | Y | N | Y | N | N | Y | Y | 8/11 (72.72%) |
| Menshw et al (2020) | Y | Y | Y | | Y | Y | | Y | Y | Y | Y | Y | Y | | Y | Y | Y | Y | Y | Y | Y | Y | Y | Y | Y | 11/11 (100%) |
| Sidamo et al (2017) | Y | Y | Y | | Y | Y | | Y | N | N | N | N | Y | | Y | Y | Y | Y | Y | N | N | N | N | Y | Y | 7/11 (63.64%) |
| Haile(2021) | Y | Y | Y | | Y | Y | | Y | U | U | U | N | Y | | Y | Y | Y | Y | Y | N | Y | N | N | Y | Y | 8/11 (72.72%) |
| Alebel et al (2020) | Y | Y | Y | | Y | Y | | Y | Y | Y | Y | Y | Y | | Y | Y | Y | Y | Y | Y | Y | Y | Y | Y | Y | 11/11 (100%) |
| Koye et al (2012) | Y | Y | Y | | Y | Y | | Y | Y | Y | Y | Y | Y | | Y | Y | Y | Y | Y | Y | U | U | Y | Y | Y | 10/11 (90.9%) |
| Gebremedihn et al (2013) | Y | Y | Y | | Y | Y | | Y | Y | Y | Y | Y | Y | | Y | Y | Y | Y | Y | U | Y | Y | Y | Y | Y | 10.5/11 (95.4%) |
| Gemech etal (2022) | Y | Y | Y | | Y | Y | | Y | Y | Y | Y | Y | Y | | Y | Y | Y | Y | Y | Y | U | U | Y | Y | Y | 10/11 (90.9%) |
| Tagesse et al (2020) | Y | Y | Y | | Y | Y | | Y | N | N | N | N | Y | | Y | Y | Y | Y | Y | N | N | N | N | Y | Y | 7/11 (63.64%) |
| Fisiha et al (2020) | Y | Y | Y | | Y | Y | | Y | Y | Y | Y | Y | Y | | Y | Y | Y | Y | Y | U | N | U | N | Y | Y | 9/11 (81.82%) |
| Atallel et al (2018) | Y | Y | Y | | Y | Y | | Y | Y | Y | Y | Y | Y | | Y | Y | Y | Y | Y | N | Y | N | Y | Y | Y | 10.5/11 (95.4%) |
| Biyazin et al (2022) | Y | Y | Y | | Y | Y | | Y | Y | Y | Y | Y | Y | | Y | Y | Y | Y | Y | Y | U | U | Y | Y | Y | 10/11 (90.9%) |
| Sifr et al (2021) | Y | Y | Y | | Y | Y | | Y | Y | Y | Y | Y | Y | | Y | Y | Y | Y | Y | N | N | N | N | Y | Y | 10/11 (90.9%) |
| Hibstie et al (2020) | Y | Y | Y | | Y | Y | | Y | Y | Y | Y | Y | Y | | Y | Y | Y | Y | Y | N | N | N | N | Y | Y | 10/11 (90.9%) |
| seid (2023) | Y | Y | Y | | Y | Y | | Y | U | U | U | N | Y | | Y | Y | Y | Y | Y | N | Y | N | N | Y | Y | 8/11 (72.72%) |
| Alemu et al(2022) | Y | Y | Y | | Y | Y | | Y | Y | Y | Y | Y | Y | | Y | Y | Y | Y | Y | Y | U | U | Y | Y | Y | 10/11 (90.9%) |
| Biru et al (2018) | Y | Y | Y | | Y | Y | | Y | Y | Y | Y | Y | Y | | Y | Y | Y | Y | Y | N | Y | N | Y | Y | Y | 10.5/11(95.4%) |
| Melaku et al (2017) | Y | Y | Y | | Y | Y | | Y | Y | Y | Y | Y | Y | | Y | Y | Y | Y | Y | N | Y | N | Y | Y | Y | 10/11 (90.9%) |
| Bankere et al (2022) | Y | Y | Y | | Y | Y | | Y | U | U | U | N | Y | | Y | Y | Y | Y | Y | N | Y | N | N | Y | Y | 8/11 (72.72%) |
| Fetene et al(2018) | Y | Y | Y | | Y | Y | | Y | N | N | N | N | Y | | Y | Y | Y | Y | Y | N | N | N | N | Y | Y | 7/11 (63.64%) |
