# Supplementary material for: Incidence of lost to follow up among HIV-positive children on antiretroviral therapy in Ethiopia: Systematic review and meta-analysis
Source: PLoS One. 2024 May 22;19(5):e0304239. doi: 10.1371/journal.pone.0304239 (PMC11111029; doi:10.1371/journal.pone.0304239)
Supplement: Attachment — Submitted filename: Response to reviewers final.docx [file pone.0304239.s004.docx]

**Author response to Editor and reviewers**

**Dear Editor and Reviewers,**

Thank you very much for your email dated on Feb 2, 2024incorporating the insight of the editor and reviewer’s comments. We, the authors, would like to express our gratitude to you for the insightful and constructive review that has led to the great improvement of our paper entitled **“Incidence of lost to follow up among HIV-positive children on antiretroviral therapy in Ethiopia: systematic review and meta-analysis.** “We declared that all the data underlying the results presented in the study are publicly available in Harvard Dataverse Network repository: <https://doi.org/10.7910/DVN/BQ6MU0>. We have carefully reviewed the comments given by the editor, and reviewers and revised the manuscript accordingly. Our responses are given in a point-by-point manner for the editor, and reviewers’ comments using the Author’s response to reviewer form. If you have any concerns to be addressed, we are happy to consider them.

Best regards!

**Version 1:** PONE-D-23-40483

Date: 2/16/2024

**Academic editor comments and respective author’s response**

**Editor comment 1:** 1. Please ensure that your manuscript meets PLOS ONE's style requirements, including those for file naming. The PLOS ONE style templates can be found at 
<https://journals.plos.org/plosone/s/file?id=wjVg/PLOSOne_formatting_sample_main_body.pdf> and <https://journals.plos.org/plosone/s/file?id=ba62/PLOSOne_formatting_sample_title_authors_affiliations.pdf>

**Authors Response:** Thanks very much for this comment. The whole part of the manuscript has been updated as per the PLOS ONE style templates.

**Editor comment 2.**  In the online submission form, you indicated that "The data is available at the corresponding author and may be provided upon request".All PLOS journals now require all data underlying the findings described in their manuscript to be freely available to other researchers, either 1. In a public repository, 2. Within the manuscript itself, or 3. Uploaded as supplementary information.This policy applies to all data except where public deposition would breach compliance with the protocol approved by your research ethics board. If your data cannot be made publicly available for ethical or legal reasons (e.g., public availability would compromise patient privacy), please explain your reasons on resubmission and your exemption request will be escalated for approval.

**Authors Response:** We declared that all the data underlying the results presented in the study are publicly available in Harvard Dataverse Network repository: <https://doi.org/10.7910/DVN/BQ6MU0>. **Editor comment 3** .Please include a separate caption for each figure in your manuscript.

**Authors Response:** Thanks very much dear editor for this helpful comment. A separate caption has been included for each Supporting Information files

**Editor comment 4:** Please include captions for your Supporting Information files at the end of your manuscript, and update any in-text citations to match accordingly. Please see our SupportingInformation guidelines for more information: <http://journals.plos.org/plosone/s/supporting-information>.

**Authors Response:** Thanks very much dear editor for this helpful comment. A separate caption has been included for each Supporting Information files

**Reviewer #1 comments and an author response**

We thank you very much for your big constructs and your devoting time to review our manuscript.

**Comment 1:** Lost to follow-up language has been changed to interruption in treatment in recent times. Use it since it is becoming mainstream terminology.

**Authors Response:** dear reviewer, thank you for your concern, dear reviewer, we accept the comment as comment and we also considered the terminology during the conception of the study, **however**, all of the primary studies used in this study were reported and measured as **left to follow up**, such that, 1) to be in line and consistent with those primary studies used for pooling LTFU and 2) by considering that both of the terminology have **comparable definition,** we used the terminology “**Left to follow up”** as it is in our study. Dear, reviewer, this all about our concern. Dear, reviewer, If you have any concern regarding this, we are happy to look it in our next revision**s.**

**Comment 2:** Incidence seems low: 2.79 per 100-child-year

**Authors Response:** Thanks very much for your comments, dear reviewer, yes, you are right, dear, reviewer please look the conclusions indicated on pages 2 , from lines number 42-43.

**Comments 3:** Time period of the study, Are all the studies for ages 0-14.

**Authors Response:** dear reviewer, thank you for your comments and comments were accepted corrections have made in the revised manuscript. Dear reviewer, all studies were conducted for children under 15 years that published up to November, 20/2023. Dear reviewer, all information were incorporated under heading eligibility criteria on pages 6 from lines 113-117.

**Comment 4:** Line 84-85: ‘the rise of questioning regarding the reason for taking ART medication’. The language is incorrect. I assume you mean to say: difficulty for parents to properly respond when children ask why they are taking medications.

**Authors Response:** Thanks very much for your comments, dear reviewer, comments were accepted; modification was made in the revised manuscript. Please, look from pages 4-5 from line numbers 84-89.

**Comment 5:** Lines 87-88: Remove the statement ‘Those, lost from follow-up, may interrupt the treatment or may continue taking the treatment until no longer medications are available’ as it doesn’t make any sense. Or rephrase it and convey a clear message.

**Author’s response:** dear reviewer, we thank you heartily for your constructive and supportive comments. Modification was made in the revised manuscript. Please, look from pages 5 from line numbers 89-91.

**Comment 5:** Line 98-99: Remove the statement ‘Thereby to develop a comprehensive strategic plan for 99 the identified factors at the national level.’ as it is redundant,• Line 113: you mean to say ‘1) studies conducted in Ethiopia’. Correct it, Line 125: remove ‘included’, Line 127: remove ‘for this study’ at the end of the sentence.

**Authors Response:** Thanks very much, for your insightful comments, dear reviewer, correction were made for all indicated comments in the revised manuscript.

**Comment 6:** The literature review you did and LTFU definition you provided is clear in that it describes the clinical/programmatic definition of LTFU as applied by clinicians or public health experts to follow programmatic outcome of ART treatment. If you are truly measuring LTFU as used routinely in clinical settings, the value reported should be much higher than this since you should label all children who interrupted treatment at least once for 1 month, no matter what their final follow-up status is, as LTFU. However, most studies don’t do such intensive analysis. They rather simply take final status at a certain date and calculate incidence of ‘LTFU’ at a certain date they decided to censor study participants. This underestimates the incidence of LTFU as defined in clinical settings since deaths will automatically be excluded from LTFU classification, which is incorrect for the most part. The reason is that most deaths occur at home and many ART clients are labeled as LTFU before they are traced, and their final status adjusted to ‘dead’. So, there are two options to move forward:

1. to study attrition at the end of follow-up including both death and LTFU (considering worst case scenario that all deaths were LTFU cases), OR

2. to study LTFU at the end of follow-up, which is true LTFU for clients with unknown status for more than 1 month.

Note that whichever option you choose, you need to update your operational definition of LTFU and additionally, you need to define the follow-up period to report your outcome to make the incidence rate give more sense by indicating the number of follow-up months. Most common times for reporting outcomes are: 6-month, 12-month, 18-month, 24-month, and yearly then after.

**Authors Response:** Thanks very much, dear reviewer, the comment was accepted; correction was made in the revised manuscript. Please look the operational definition of LTFU and follow-up period on page 7 from line numbers 145-148. Dear reviewer, we also indicate the person month observation, person year observation, follow up months (please look table 1).

**Dear reviewer, the segregated event (number LTFU) at** 6-month, 12-month, 18-month, 24-month, and yearly then after were not reported in the primary studies. Dear reviewer, due to this we can’t pooled the incidence of LTFU for the respective months. Dear reviewer, this is one of our limitation and we incorporate it as our limitation, please look it under heading of limitation on page 17 from lines numbers 290-292.

**Reviewer 2 comments and an author response**

Congratulations on the manuscript that addresses LTFU in Ethiopia with lessons that can be applied to other settings. The paper is well written but can be improved by addressing a couple of comments and typos and grammatical errors some of which i have pointed out. The manuscript reviews loss to follow up in studies conducted in Ethiopia. This is an important review addressing a topic important in improving treatment outcomes for children. Although it primarily focusses on studies conducted in Ethiopia, lessons have the potential to be applied elsewhere. The paper is generally well written but can be improved by addressing grammatical errors and typos throughout the manuscript and the following specific comments;

**Authors: Dear reviewer**, we thank you for your constructive comments, suggestion and devoted times. Here, we are happy to respond your comments.

**Comment 1:** Title: Should be revised to include “Ethiopia”. The revised title should read “Incidence of lost to follow up among HIV-positive children on antiretroviral treatment: systematic review and meta-analysis of studies conducted in Ethiopia”

**Authors Response:** Dear reviewer, thank you very much for your constructive comments. Corrections were made in the revised manuscript as the following “Incidence of lost to follow up among HIV-positive children on antiretroviral therapy **in Ethiopia**: systematic review and meta-analysis”.

**Comment 2:** Abstract: In conclusion, the reference t” lower incidence should probably be removed since the goal was not a comparison.

**Authors Response:** Dear reviewer, thank you very much for your constructive comments. Corrections were made in the revised manuscript indicated on page 2 from lines 42-43.

**Comment 3:** Background: Generally, gives the context. There are a few grammatical errors that should be removed. Live 67: end of the sentence should be “died from HIV related disease”. Line 74-78 should be revised to clarify the 95-95-95 goals are not only for children but all PLHIV.

**Authors Response:** Thanks very much for your constructive comments, dear reviewer. Correction was made in the revised manuscript as per your suggestion.

**Comment 4:** Line 85, 88, 89: have grammatical errors. Line 95: replace “predicting” with “establishing”. Line 115 has a typo.

**Authors Response:** Thanks very much for your insightful comments, dear reviewer; we accepted your comment. Modification was made in the revised manuscript indicated from pages 4-5, from lines 84-91.

**Comment 5:** Methods: Well written. Line 108 explain why “ADIS” was included as a search term, Line 113, replace “connected” with “conducted”

**Authors Response:** Dear reviewer, thank you very much for your constructive comments and correction. Type error was corrected. We used “ADIS” as searching term because of to incorporate articles that may state AIDS as term in the title or topic. Dear reviewer, it has no impact on our searching but it increase the inclusiveness of our searching.

**Comment 6:** Statistical analysis: Not clear what this phrase means “The data synthesis was done via tabulating”.

**Authors Response:** Thanks very much for your critical insight, dear reviewer, this edition problem and it was removed from the manuscript.

Comments 7: Please define the was HIV disease stage was assessed in the study as well as measures of adherence to ART.

**Authors Response:** dear reviewer, Thanks very much for your critical insight, dear reviewer, this edition problem and it was removed from the manuscript. Operational definition was incorporated in the revised manuscript, please look from pages 7-8, from line numbers 145-154.

**Comment 8:** Line 161: should be results not result. Results are well summarized, tables and figures are well labeled. Table 2 is difficult to understand. Line 168 should be “titles and abstracts”

**Authors Response:** Dear reviewer, Thank you for your corrective comments. Corrections were made in the revised manuscript, please, look on pages 11, from lines numbers 206-207.

**Comment 9:** Line 200-201- correct statement. Did you mean advanced HIV disease? Please explain how HIV disease staging was assessed in methods since all children 5 and below are considered to have advanced HIV disease according to WHO

Dear, reviewer, Thank you for your constructive comments. Comments are accepted. Corrections were made in the revised manuscript. Please, look on page 7 from lines 149-151

Discussion

**Comment 9: Line** 221. Delete ART “follow up”. Line 223, replace “finding” with “incidence”. Line 223-230, the authors compared the pooled incidence with reported incidence from single studies. Not sure that this is a fair comparison.

**Authors Response:** dear, reviewer, thank you for your constructive comments. Dear reviewer, we absolutely accept your concern, yes you are right. Dear reviewer, though we browsed for systematic and meta-analysis studies conducted on Incidence of lost to follow up among children, we can’t access systematic and meta-analysis studies conducted in other countries. Because of this we are obligated to use primary study for comparing our pooled estimate of Incidence of lost to follow. However, some of those studies were nationwide and were representative of the countries.

**Comment 10:** Line 248-9: statement not clear since people get opportunistic infections because they have lower immunity. Requires editing to make scientific sense. OIs do not reduce the CD4 rather they occur or manifest in the setting of reduced immunity.

**Authors Response:** dear, reviewer, thank you for your constructive comments, comments were accepted, corrections were made in the revised manuscript, please look on page 15 from lines 251-257.

**Reviewer 3 comments and an author response**

Yes, the right statistical analysis for a systematic review was performed appropriately and rigorously.
3. Yes, the authors made all data underlying the findings in the manuscript fully available
4. Yes, the manuscript was presented in an intelligible fashion and written in standard English.

**Authors: Dear reviewer**, we thank you for your constructive comments, suggestion and devoted times. Here, we are happy to respond your comments.

**Comments 1:** However, there were lots of minor typos. Thoroughly review is needed.

For **example, in the abstract Background section**

- Line 7 - change "variations were seen between the studies" to "variations were seen among the studies"
- Line 8 - change incidence of lost follow-up to "incidence of lost to follow-up". Insert to between lost and follow up

**Author’s response:** Dear reviewer, thank you very much for your constructive comments and correction. Corrections were made in the revised manuscript as per your suggestion.

**Comments 2: Method -**

- **Line 1 - change "we searched" to "We searched" with capital W**
- **Line 5 - change "Fixed effect models" to "fixed effect models" with a small case letter 'f'**
- **Similar errors were observed in the abstract. Do correct.**

**Author’s response:** Dear reviewer, thank you very much for your constructive comments and correction. Corrections were made in the revised manuscript as per your suggestion.

**Comments 3:** Line 69, change 'different' effort to 'several or much or various or multiple' effort
Line 71, change "By" to small case b'' as in by.

**Author’s response:** Dear reviewer, thank you very much for your constructive comments and correction. Corrections were made in the revised manuscript as per your suggestion, please look on page 4 on line number 68.

**Comments:** It will be okay to define 'fair or poor ART treatment'

**Author’s response:** Dear reviewer, thank you very much for your constructive comments and suggestion. Comment were accepted and corrections were made in the revised manuscript as per your suggestion, please look on page 8 from line numbers 152-155.
