# Supplementary material for: Incidence of lost to follow up among HIV-positive children on antiretroviral therapy in Ethiopia: Systematic review and meta-analysis
Source: PLoS One. 2024 May 22;19(5):e0304239. doi: 10.1371/journal.pone.0304239 (PMC11111029; doi:10.1371/journal.pone.0304239)
Supplement: Attachment — Submitted filename: Incidence of lost to follow up among HIV_min rev.docx [file pone.0304239.s005.docx]

**Incidence of lost to follow up among HIV-positive children on antiretroviral therapy in**

**Ethiopia: systematic review and meta-analysis**

**General Comments and suggestions**

The author may revise the English language throughout the manuscript: punctuation, grammar… may be rechecked. Revising the manuscript against the journal requirement; specifically, the way reference is listed and written be looked into.

**Specific comments and suggestions**

#32, 104, Change ‘November 20/2023’ to November 20, 2023

\# 42, 104, 116, 144, 168: Change ‘Left to follow up’ and ‘loss to follow-up’ to lost to follow-up and make consistent throughout.

#43: why “Counseling’ started with capital letter?

#65-67: could be two separate sentences. You may put a full stop after ‘… end of 2022’.

\# 91-92, 187: check if you need to add ’ after ART initiation’ at end the sentence and the title in # 187. Note there a are a lot of LTFU incidence evidence among HIV children, but fewer while after they put on ART.

#122,123: ‘… three 123 authors (GF, ZA, GM, and MS).’ Check if these are these three or four.

\# 123: you may replace ‘between’ with ‘among’

#124: “The each study…’’ what does this mean? Or you may replace ‘The’ with ‘In’

#133: ‘Twenty-four studies...’ could be replaced by ‘twenty-four studies...”

#159: ‘’… 25, 50, and 75%,’’ make the % uniform to all numbers.

#242: check whether “Waster Kenya” is right?

#181-288: (limitation): consider revising, summarize this section. May omit numbers 1-5.

Reference: Try to revise the write up of the reference per the journal requirement.
