# Supplementary material for: Incidence of lost to follow up among HIV-positive children on antiretroviral therapy in Ethiopia: Systematic review and meta-analysis
Source: PLoS One. 2024 May 22;19(5):e0304239. doi: 10.1371/journal.pone.0304239 (PMC11111029; doi:10.1371/journal.pone.0304239)
Supplement: Attachment — Submitted filename: Response to reviewers final1.docx [file pone.0304239.s006.docx]

**Author response to Editor and reviewers**

**Dear Editor and Reviewers,**

Thank you very much for your email dated on May 2, 2024 incorporating the insight of the editor and reviewer’s comments. We, the authors, would like to express our gratitude to you for the insightful and constructive review that has led to the great improvement of our paper entitled **“Incidence of lost to follow up among HIV-positive children on antiretroviral therapy in Ethiopia: systematic review and meta-analysis.** “We declared that all the data underlying the results presented in the study are publicly available in Harvard Dataverse Network repository: <https://doi.org/10.7910/DVN/BQ6MU0>. We have carefully reviewed the comments given by the editor, and reviewers and revised the manuscript accordingly. Our responses are given in a point-by-point manner for the editor, and reviewers’ comments using the Author’s response to reviewer form. If you have any concerns to be addressed, we are happy to consider them.

Best regards!

**Version 2:** PONE-D-23-40483

Date: May 3, 2024

**Academic editor comments and respective author’s response**

We thank you very much for your big constructs and your constructive comments

**Editor comment 1:** The author may revise the English language throughout the manuscript: punctuation, grammar… may be rechecked. Revising the manuscript against the journal requirement; specifically, the way reference is listed and written be looked into.

**Authors Response:** Thanks very much for this comment. The punctuation, grammar was rigorously rechecked

**Editor comment 2.**  #32, 104, Change ‘November 20/2023’ to November 20, 2023

**Authors Response:** dear editors, thank you for your corrective comments. Correction was made in the revised manuscript.

**Editors comments:** # 42, 104, 116, 144, 168: Change ‘Left to follow up’ and ‘loss to follow-up’ to lost to follow-up and make consistent throughout.

**Authors Response:** dear editors, thank you for your corrective comments. Correction was made in the throughout the revised manuscript

**Editor comment 4:** #43: why “Counseling’ started with capital letter?.

**Authors Response:** dear editors, thank you for your corrective comments. Correction was made in the revised manuscript.

**Editor’s comment:** #65-67: could be two separate sentences. You may put a full stop after ‘… end of 2022’.

**Authors Response:** dear editors, thank you for your corrective comments. Correction was made in the revised manuscript.

**Editors comments:** # 91-92, 187: check if you need to add ’ after ART initiation’ at end the sentence and the title in # 187. Note there a are a lot of LTFU incidence evidence among HIV children, but fewer while after they put on ART.

**Authors Response:** dear editors, thank you for your corrective comments. Correction was made in the throughout the revised manuscript

**Editor comments :** #122,123: ‘… three 123 authors (GF, ZA, GM, and MS).’ Check if these are these three or four.# 123: you may replace ‘between’ with ‘among’

**Authors Response:** dear editors, thank you for your corrective comments. Correction was made in the revised manuscript.

**Editor comments:** #124: “The each study…’’ what does this mean? Or you may replace ‘The’ with ‘In’

**Authors Response:** dear editors, thank you for your corrective comments. Correction was made in the revised manuscript.

**Editor comments:** #133: ‘Twenty-four studies...’ could be replaced by ‘twenty-four studies...”

**Authors Response:** dear editors, thank you for your corrective comments. Correction was made in the revised manuscript.

Editors comments: #159: ‘’… 25, 50, and 75%,’’ make the % uniform to all numbers.

**Authors Response:** dear editors, thank you for your corrective comments. Correction was made in the revised manuscript.

Editor comments: #242: check whether “Waster Kenya” is right?

**Authors Response:** dear editors, thank you for your corrective comments. Correction was made in the revised manuscript.

**Authors Response:** dear editors, thank you for your corrective comments. Correction was made in the revised manuscript.

Editor comments : #181-288: (limitation): consider revising, summarize this section. May omit numbers 1-5.

**Authors Response:** dear editors, thank you for your corrective comments. Correction was made in the revised manuscript.

**Editor comments Reference**: Try to revise the write up of the reference per the journal requirement.:

Please review your reference list to ensure that it is complete and correct. If you have cited papers that have been retracted, please include the rationale for doing so in the manuscript text, or remove these references and replace them with relevant current references. Any changes to the reference list should be mentioned in the rebuttal letter that accompanies your revised manuscript. If you need to cite a retracted article, indicate the article’s retracted status in the References list and also include a citation and full reference for the retraction notice.

**Authors Response:** dear editors, thank you for your constructive comments. Dear editors, we didn’t used any retracted and we made revision on the write up of the reference in the revised manuscript.

**Reviewer #1 comments and an author response**

We thank you very much for your big constructs and your devoting time to review our manuscript.

**Comment 1:** Reviewer #1: Most of the comments have been addressed. However, the fact that LTFU incidence is low doesn't mean that retention was optimal. This is because the operational definition of LTFU is re-defined as those who interrupted treatment and has not been classified as dead or transferred out. This means, LTFU incidence in this meta-analysis measures cases whose tracing outcome remains unknown. This is a positive finding for the tracing mechanism put in place since deaths, and transfer out cases are being identified and reported. However, the way the paper is written, it seems to suggest LTFU was "generally low" for children with HIV. This is far from reality since retention among children with HIV is far lower that adults with HIV and adult retention at 12 months has not been more than 85% in many cases.

In order to avoid this confusion, clearly state this in the discussion section that

1. LTFU means those with unknown tracing outcome

2. LTFU is not a reflection of improved retention in care rather the improved tracing and reporting mechanism

**Authors Response:** dear reviewer, thank you for your concern, dear reviewer, and correction was made in the revised manuscript, please, look on page 14 from lines numbers 242 to 246

**Comment 2:** Finally, make sure to proof read the paper since words like 'ADIS' still remain in the revised version, which should have been edited out as per reviewers comment.

**Authors Response:** dear reviewer, thank you for your corrective comments. Correction was made in the revised manuscript.
