# Supplementary material for: Incidence of lost to follow up among HIV-positive children on antiretroviral therapy in Ethiopia: Systematic review and meta-analysis
Source: PLoS One. 2024 May 22;19(5):e0304239. doi: 10.1371/journal.pone.0304239 (PMC11111029; doi:10.1371/journal.pone.0304239)
Supplement: Peer review history

# Peer review: Decision Letter 0

**DOI:** 10.1371/journal.pone.0304239.r001

**Authors:** Zewdu Gashu Dememew

1 Feb 2024

PONE-D-23-40483Incidence of lost to follow up among HIV-positive children on antiretroviral treatment: systematic review and meta-analysis.PLOS ONE

Dear Dr. Girma,

Thank you for submitting your manuscript to PLOS ONE. After careful consideration, we feel that it has merit but does not fully meet PLOS ONE’s publication criteria as it currently stands. Therefore, we invite you to submit a revised version of the manuscript that addresses the points raised during the review process.

Please submit your revised manuscript by Mar 17 2024 11:59PM. If you will need more time than this to complete your revisions, please reply to this message or contact the journal office at plosone@plos.org. When you're ready to submit your revision, log on to https://www.editorialmanager.com/pone/ and select the 'Submissions Needing Revision' folder to locate your manuscript file.

Please include the following items when submitting your revised manuscript:

- A rebuttal letter that responds to each point raised by the academic editor and reviewer(s). You should upload this letter as a separate file labeled 'Response to Reviewers'.
- A marked-up copy of your manuscript that highlights changes made to the original version. You should upload this as a separate file labeled 'Revised Manuscript with Track Changes'.
- An unmarked version of your revised paper without tracked changes. You should upload this as a separate file labeled 'Manuscript'.

If you would like to make changes to your financial disclosure, please include your updated statement in your cover letter. Guidelines for resubmitting your figure files are available below the reviewer comments at the end of this letter.

If applicable, we recommend that you deposit your laboratory protocols in protocols.io to enhance the reproducibility of your results. Protocols.io assigns your protocol its own identifier (DOI) so that it can be cited independently in the future. For instructions see: https://journals.plos.org/plosone/s/submission-guidelines#loc-laboratory-protocols. Additionally, PLOS ONE offers an option for publishing peer-reviewed Lab Protocol articles, which describe protocols hosted on protocols.io. Read more information on sharing protocols at https://plos.org/protocols?utm_medium=editorial-email&utm_source=authorletters&utm_campaign=protocols.

We look forward to receiving your revised manuscript.

Kind regards,

Zewdu Gashu Dememew, M.D, PhD

Academic Editor

PLOS ONE

Journal Requirements:

When submitting your revision, we need you to address these additional requirements.

1. Please ensure that your manuscript meets PLOS ONE's style requirements, including those for file naming. The PLOS ONE style templates can be found at 

https://journals.plos.org/plosone/s/file?id=wjVg/PLOSOne_formatting_sample_main_body.pdf and 

https://journals.plos.org/plosone/s/file?id=ba62/PLOSOne_formatting_sample_title_authors_affiliations.pdf

2. In the online submission form, you indicated that "The data is available at the corresponding author and may be provided upon request".

All PLOS journals now require all data underlying the findings described in their manuscript to be freely available to other researchers, either 1. In a public repository, 2. Within the manuscript itself, or 3. Uploaded as supplementary information.

This policy applies to all data except where public deposition would breach compliance with the protocol approved by your research ethics board. If your data cannot be made publicly available for ethical or legal reasons (e.g., public availability would compromise patient privacy), please explain your reasons on resubmission and your exemption request will be escalated for approval.

3. Please include a separate caption for each figure in your manuscript.

4. Please include captions for your Supporting Information files at the end of your manuscript, and update any in-text citations to match accordingly. Please see our Supporting Information guidelines for more information: http://journals.plos.org/plosone/s/supporting-information.

[Note: HTML markup is below. Please do not edit.]

Reviewers' comments:

Reviewer's Responses to Questions

**Comments to the Author**

1. Is the manuscript technically sound, and do the data support the conclusions?

The manuscript must describe a technically sound piece of scientific research with data that supports the conclusions. Experiments must have been conducted rigorously, with appropriate controls, replication, and sample sizes. The conclusions must be drawn appropriately based on the data presented. 

Reviewer #1: Partly

Reviewer #2: Partly

Reviewer #3: Yes

**********

2. Has the statistical analysis been performed appropriately and rigorously? 

Reviewer #1: Yes

Reviewer #2: Yes

Reviewer #3: Yes

**********

3. Have the authors made all data underlying the findings in their manuscript fully available?

The PLOS Data policy requires authors to make all data underlying the findings described in their manuscript fully available without restriction, with rare exception (please refer to the Data Availability Statement in the manuscript PDF file). The data should be provided as part of the manuscript or its supporting information, or deposited to a public repository. For example, in addition to summary statistics, the data points behind means, medians and variance measures should be available. If there are restrictions on publicly sharing data—e.g. participant privacy or use of data from a third party—those must be specified.

Reviewer #1: Yes

Reviewer #2: Yes

Reviewer #3: Yes

**********

4. Is the manuscript presented in an intelligible fashion and written in standard English?

PLOS ONE does not copyedit accepted manuscripts, so the language in submitted articles must be clear, correct, and unambiguous. Any typographical or grammatical errors should be corrected at revision, so please note any specific errors here.

Reviewer #1: No

Reviewer #2: Yes

Reviewer #3: Yes

**********

5. Review Comments to the Author

Please use the space provided to explain your answers to the questions above. You may also include additional comments for the author, including concerns about dual publication, research ethics, or publication ethics. (Please upload your review as an attachment if it exceeds 20,000 characters)

Reviewer #1: • Lost to follow-up language has been changed to interruption in treatment in recent times. Use it since it is becoming mainstream terminology.

• Line 36: Incidence seems low: 2.79 per 100-child-year

• Time period of the study

• Are all the studies for ages 0-14?

• Line 84-85: ‘the rise of questioning regarding the reason for taking ART medication’. The language is incorrect. I assume you mean to say: difficulty for parents to properly respond when children ask why they are taking medications.

• Lines 87-88: Remove the statement ‘Those, lost from follow-up, may interrupt the treatment or may continue taking the treatment until no longer medications are available’ as it doesn’t make any sense. Or rephrase it and convey a clear message.

• Line 98-99: Remove the statement ‘Thereby to develop a comprehensive strategic plan for 99 the identified factors at the national level.’ as it is redundant.

• Line 113: you mean to say ‘1) studies conducted in Ethiopia’. Correct it.

• Line 125: remove ‘included’.

• Line 127: remove ‘for this study’ at the end of the sentence.

• The literature review you did and LTFU definition you provided is clear in that it describes the clinical/programmatic definition of LTFU as applied by clinicians or public health experts to follow programmatic outcome of ART treatment. If you are truly measuring LTFU as used routinely in clinical settings, the value reported should be much higher than this since you should label all children who interrupted treatment at least once for 1 month, no matter what their final follow-up status is, as LTFU. However, most studies don’t do such intensive analysis. They rather simply take final status at a certain date and calculate incidence of ‘LTFU’ at a certain date they decided to censor study participants. This underestimates the incidence of LTFU as defined in clinical settings since deaths will automatically be excluded from LTFU classification, which is incorrect for the most part. The reason is that most deaths occur at home and many ART clients are labeled as LTFU before they are traced, and their final status adjusted to ‘dead’. So, there are two options to move forward:

1. to study attrition at the end of follow-up including both death and LTFU (considering worst case scenario that all deaths were LTFU cases), OR

2. to study LTFU at the end of follow-up, which is true LTFU for clients with unknown status for more than 1 month.

Note that whichever option you choose, you need to update your operational definition of LTFU and additionally, you need to define the follow-up period to report your outcome to make the incidence rate give more sense by indicating the number of follow-up months. Most common times for reporting outcomes are: 6-month, 12-month, 18-month, 24-month, and yearly then after.

• Language needs to be edited by a professional to make it more readable for all audiences including international readers.

Reviewer #2: Congratulations on the manuscript that addresses LTFU in Ethiopia with lessons that can be applied to other settings. The paper is well written but can be improved by addressing a couple of comments and typos and grammatical errors some of which i have pointed out.

Review: Incidence of lost to follow up among HIV-positive children on antiretroviral treatment:

systematic review and meta-analysis

The manuscript reviews loss to follow up in studies conducted in Ethiopia. This is an important review addressing a topic important in improving treatment outcomes for children. Although it primarily focusses on studies conducted in Ethiopia, lessons have the potential to be applied elsewhere.

The paper is generally well written but can be improved by addressing grammatical errors and typos throughout the manuscript and the following specific comments;

Specific comments:

Title: Should be revised to include “Ethiopia”. The revised title should read “Incidence of lost to follow up among HIV-positive children on antiretroviral treatment: systematic review and meta-analysis of studies conducted in Ethiopia”

Abstract: In conclusion, the reference to lower incidence should probably be removed since the goal was not a comparison.

Background: Generally, gives the context. There are a few grammatical errors that should be removed. Live 67: end of the sentence should be “died from HIV related disease”. Line 74-78 should be revised to clarify the 95-95-95 goals are not only for children but all PLHIV.

Line 85, 88, 89: have grammatical errors. Line 95: replace “predicting” with “establishing”. Line 115 has a typo

Methods: Well written. Line 108 explain why “ADIS” was included as a search term,Line 113, replace “connected” with “conducted”

Statistical analysis: Not clear what this phrase means “The data synthesis was done via tabulating”. Please define the was HIV disease stage was assessed in the study as well as measures of adherence to ART.

Results

Line 161: should be results not result. Results are well summarized, tables and figures are well labeled. Table 2 is difficult to understand. Line 168 should be “titles and abstracts”

Line 200-201- correct statement. Did you mean advanced HIV disease. Please explain how HIV disease staging was assessed in methods since all children 5 and below are considered to have advanced HIV disease according to WHO.

Discussion: discusses the findings.

Line 221. Delete ART “follow up”. Line 223, replace “finding” with “incidence”. Line 223-230, the authors compared the pooled incidence with reported incidence from single studies. Not sure that this is a fair comparison.

Line 248-9: statement not clear since people get opportunistic infections because they have lower immunity. Requires editing to make scientific sense. OIs do not reduce the CD4 rather they occur or manifest in the setting of reduced immunity.

Reviewer #3: 1. Yes, it is technically sound.

2. Yes, the right statistical analysis for a systematic review was performed appropriately and rigorously.

3. Yes, the authors made all data underlying the findings in the manuscript fully available

4. Yes, the manuscript was presented in an intelligible fashion and written in standard English.

However, there were lots of minor typos. Thoroughly review is needed.

For example,

In the abstract

Background section -

Line 7 - change "variations were seen between the studies" to "variations were seen among the studies"

Line 8 - change incidence of lost follow-up to "incidence of lost to follow-up". Insert to between lost and follow up

Method -

Line 1 - change "we searched" to "We searched" with capital W

Line 5 - change "Fixed effect models" to "fixed effect models" with a small case letter 'f'

Similar errors were observed in the abstract. Do correct.

Line 69, change 'different' effort to 'several or much or various or multiple' effort

Line 71, change "By" to small case b'' as in by.

It will be okay to define 'fair or poor ART treatment'

**********

6. PLOS authors have the option to publish the peer review history of their article (what does this mean?). If published, this will include your full peer review and any attached files.

If you choose “no”, your identity will remain anonymous but your review may still be made public.

**Do you want your identity to be public for this peer review?** For information about this choice, including consent withdrawal, please see our Privacy Policy.

Reviewer #1: **Yes: **Kesetebirhan Delele Yirdaw

Reviewer #2: No

Reviewer #3: No

**********

[NOTE: If reviewer comments were submitted as an attachment file, they will be attached to this email and accessible via the submission site. Please log into your account, locate the manuscript record, and check for the action link "View Attachments". If this link does not appear, there are no attachment files.]

While revising your submission, please upload your figure files to the Preflight Analysis and Conversion Engine (PACE) digital diagnostic tool, https://pacev2.apexcovantage.com/. PACE helps ensure that figures meet PLOS requirements. To use PACE, you must first register as a user. Registration is free. Then, login and navigate to the UPLOAD tab, where you will find detailed instructions on how to use the tool. If you encounter any issues or have any questions when using PACE, please email PLOS at figures@plos.org. Please note that Supporting Information files do not need this step.

# Author comment: Author response to Decision Letter 0

**DOI:** 10.1371/journal.pone.0304239.r002

19 Feb 2024

Academic editor comments and respective author’s response

Editor comment 1: 1. Please ensure that your manuscript meets PLOS ONE's style requirements, including those for file naming. The PLOS ONE style templates can be found at 

https://journals.plos.org/plosone/s/file?id=wjVg/PLOSOne_formatting_sample_main_body.pdf and https://journals.plos.org/plosone/s/file?id=ba62/PLOSOne_formatting_sample_title_authors_affiliations.pdf

Authors Response: Thanks very much for this comment. The whole part of the manuscript has been updated as per the PLOS ONE style templates.

Editor comment 2. In the online submission form, you indicated that "The data is available at the corresponding author and may be provided upon request".All PLOS journals now require all data underlying the findings described in their manuscript to be freely available to other researchers, either 1. In a public repository, 2. Within the manuscript itself, or 3. Uploaded as supplementary information.This policy applies to all data except where public deposition would breach compliance with the protocol approved by your research ethics board. If your data cannot be made publicly available for ethical or legal reasons (e.g., public availability would compromise patient privacy), please explain your reasons on resubmission and your exemption request will be escalated for approval.

Authors Response: We declared that all the data underlying the results presented in the study are publicly available in Harvard Dataverse Network repository: https://doi.org/10.7910/DVN/BQ6MU0. Editor comment 3 .Please include a separate caption for each figure in your manuscript.

Authors Response: Thanks very much dear editor for this helpful comment. A separate caption has been included for each Supporting Information files 

Editor comment 4: Please include captions for your Supporting Information files at the end of your manuscript, and update any in-text citations to match accordingly. Please see our SupportingInformation guidelines for more information: http://journals.plos.org/plosone/s/supporting-information.

 Authors Response: Thanks very much dear editor for this helpful comment. A separate caption has been included for each Supporting Information files 

Reviewer #1 comments and an author response

We thank you very much for your big constructs and your devoting time to review our manuscript. 

Comment 1: Lost to follow-up language has been changed to interruption in treatment in recent times. Use it since it is becoming mainstream terminology.

Authors Response: dear reviewer, thank you for your concern, dear reviewer, we accept the comment as comment and we also considered the terminology during the conception of the study, however, all of the primary studies used in this study were reported and measured as left to follow up, such that, 1) to be in line and consistent with those primary studies used for pooling LTFU and 2) by considering that both of the terminology have comparable definition, we used the terminology “Left to follow up” as it is in our study. Dear, reviewer, this all about our concern. Dear, reviewer, If you have any concern regarding this, we are happy to look it in our next revisions.

Comment 2: Incidence seems low: 2.79 per 100-child-year 

Authors Response: Thanks very much for your comments, dear reviewer, yes, you are right, dear, reviewer please look the conclusions indicated on pages 2 , from lines number 42-43. 

Comments 3: Time period of the study, Are all the studies for ages 0-14.

Authors Response: dear reviewer, thank you for your comments and comments were accepted corrections have made in the revised manuscript. Dear reviewer, all studies were conducted for children under 15 years that published up to November, 20/2023. Dear reviewer, all information were incorporated under heading eligibility criteria on pages 6 from lines 113-117.

Comment 4: Line 84-85: ‘the rise of questioning regarding the reason for taking ART medication’. The language is incorrect. I assume you mean to say: difficulty for parents to properly respond when children ask why they are taking medications.

Authors Response: Thanks very much for your comments, dear reviewer, comments were accepted; modification was made in the revised manuscript. Please, look from pages 4-5 from line numbers 84-89.

Comment 5: Lines 87-88: Remove the statement ‘Those, lost from follow-up, may interrupt the treatment or may continue taking the treatment until no longer medications are available’ as it doesn’t make any sense. Or rephrase it and convey a clear message.

Author’s response: dear reviewer, we thank you heartily for your constructive and supportive comments. Modification was made in the revised manuscript. Please, look from pages 5 from line numbers 89-91.

Comment 5: Line 98-99: Remove the statement ‘Thereby to develop a comprehensive strategic plan for 99 the identified factors at the national level.’ as it is redundant,• Line 113: you mean to say ‘1) studies conducted in Ethiopia’. Correct it, Line 125: remove ‘included’, Line 127: remove ‘for this study’ at the end of the sentence.

Authors Response: Thanks very much, for your insightful comments, dear reviewer, correction were made for all indicated comments in the revised manuscript.

Comment 6: The literature review you did and LTFU definition you provided is clear in that it describes the clinical/programmatic definition of LTFU as applied by clinicians or public health experts to follow programmatic outcome of ART treatment. If you are truly measuring LTFU as used routinely in clinical settings, the value reported should be much higher than this since you should label all children who interrupted treatment at least once for 1 month, no matter what their final follow-up status is, as LTFU. However, most studies don’t do such intensive analysis. They rather simply take final status at a certain date and calculate incidence of ‘LTFU’ at a certain date they decided to censor study participants. This underestimates the incidence of LTFU as defined in clinical settings since deaths will automatically be excluded from LTFU classification, which is incorrect for the most part. The reason is that most deaths occur at home and many ART clients are labeled as LTFU before they are traced, and their final status adjusted to ‘dead’. So, there are two options to move forward:

1. to study attrition at the end of follow-up including both death and LTFU (considering worst case scenario that all deaths were LTFU cases), OR

2. to study LTFU at the end of follow-up, which is true LTFU for clients with unknown status for more than 1 month.

Note that whichever option you choose, you need to update your operational definition of LTFU and additionally, you need to define the follow-up period to report your outcome to make the incidence rate give more sense by indicating the number of follow-up months. Most common times for reporting outcomes are: 6-month, 12-month, 18-month, 24-month, and yearly then after.

Authors Response: Thanks very much, dear reviewer, the comment was accepted; correction was made in the revised manuscript. Please look the operational definition of LTFU and follow-up period on page 7 from line numbers 145-148. Dear reviewer, we also indicate the person month observation, person year observation, follow up months (please look table 1).

Dear reviewer, the segregated event (number LTFU) at 6-month, 12-month, 18-month, 24-month, and yearly then after were not reported in the primary studies. Dear reviewer, due to this we can’t pooled the incidence of LTFU for the respective months. Dear reviewer, this is one of our limitation and we incorporate it as our limitation, please look it under heading of limitation on page 17 from lines numbers 290-292.

Reviewer 2 comments and an author response

Congratulations on the manuscript that addresses LTFU in Ethiopia with lessons that can be applied to other settings. The paper is well written but can be improved by addressing a couple of comments and typos and grammatical errors some of which i have pointed out. The manuscript reviews loss to follow up in studies conducted in Ethiopia. This is an important review addressing a topic important in improving treatment outcomes for children. Although it primarily focusses on studies conducted in Ethiopia, lessons have the potential to be applied elsewhere. The paper is generally well written but can be improved by addressing grammatical errors and typos throughout the manuscript and the following specific comments;

Authors: Dear reviewer, we thank you for your constructive comments, suggestion and devoted times. Here, we are happy to respond your comments.

Comment 1: Title: Should be revised to include “Ethiopia”. The revised title should read “Incidence of lost to follow up among HIV-positive children on antiretroviral treatment: systematic review and meta-analysis of studies conducted in Ethiopia”

Authors Response: Dear reviewer, thank you very much for your constructive comments. Corrections were made in the revised manuscript as the following “Incidence of lost to follow up among HIV-positive children on antiretroviral therapy in Ethiopia: systematic review and meta-analysis”.

Comment 2: Abstract: In conclusion, the reference t” lower incidence should probably be removed since the goal was not a comparison.

Authors Response: Dear reviewer, thank you very much for your constructive comments. Corrections were made in the revised manuscript indicated on page 2 from lines 42-43.

Comment 3: Background: Generally, gives the context. There are a few grammatical errors that should be removed. Live 67: end of the sentence should be “died from HIV related disease”. Line 74-78 should be revised to clarify the 95-95-95 goals are not only for children but all PLHIV. 

Authors Response: Thanks very much for your constructive comments, dear reviewer. Correction was made in the revised manuscript as per your suggestion.

Comment 4: Line 85, 88, 89: have grammatical errors. Line 95: replace “predicting” with “establishing”. Line 115 has a typo.

Authors Response: Thanks very much for your insightful comments, dear reviewer; we accepted your comment. Modification was made in the revised manuscript indicated from pages 4-5, from lines 84-91. 

Comment 5: Methods: Well written. Line 108 explain why “ADIS” was included as a search term, Line 113, replace “connected” with “conducted”

Authors Response: Dear reviewer, thank you very much for your constructive comments and correction. Type error was corrected. We used “ADIS” as searching term because of to incorporate articles that may state AIDS as term in the title or topic. Dear reviewer, it has no impact on our searching but it increase the inclusiveness of our searching.

Comment 6: Statistical analysis: Not clear what this phrase means “The data synthesis was done via tabulating”. 

Authors Response: Thanks very much for your critical insight, dear reviewer, this edition problem and it was removed from the manuscript.

Comments 7: Please define the was HIV disease stage was assessed in the study as well as measures of adherence to ART. 

Authors Response: dear reviewer, Thanks very much for your critical insight, dear reviewer, this edition problem and it was removed from the manuscript. Operational definition was incorporated in the revised manuscript, please look from pages 7-8, from line numbers 145-154.

Comment 8: Line 161: should be results not result. Results are well summarized, tables and figures are well labeled. Table 2 is difficult to understand. Line 168 should be “titles and abstracts”

Authors Response: Dear reviewer, Thank you for your corrective comments. Corrections were made in the revised manuscript, please, look on pages 11, from lines numbers 206-207.

Comment 9: Line 200-201- correct statement. Did you mean advanced HIV disease? Please explain how HIV disease staging was assessed in methods since all children 5 and below are considered to have advanced HIV disease according to WHO

Dear, reviewer, Thank you for your constructive comments. Comments are accepted. Corrections were made in the revised manuscript. Please, look on page 7 from lines 149-151 

Discussion

Comment 9: Line 221. Delete ART “follow up”. Line 223, replace “finding” with “incidence”. Line 223-230, the authors compared the pooled incidence with reported incidence from single studies. Not sure that this is a fair comparison.

Authors Response: dear, reviewer, thank you for your constructive comments. Dear reviewer, we absolutely accept your concern, yes you are right. Dear reviewer, though we browsed for systematic and meta-analysis studies conducted on Incidence of lost to follow up among children, we can’t access systematic and meta-analysis studies conducted in other countries. Because of this we are obligated to use primary study for comparing our pooled estimate of Incidence of lost to follow. However, some of those studies were nationwide and were representative of the countries. 

Comment 10: Line 248-9: statement not clear since people get opportunistic infections because they have lower immunity. Requires editing to make scientific sense. OIs do not reduce the CD4 rather they occur or manifest in the setting of reduced immunity.

Authors Response: dear, reviewer, thank you for your constructive comments, comments were accepted, corrections were made in the revised manuscript, please look on page 15 from lines 251-257. 

Reviewer 3 comments and an author response

Yes, the right statistical analysis for a systematic review was performed appropriately and rigorously.

3. Yes, the authors made all data underlying the findings in the manuscript fully available

4. Yes, the manuscript was presented in an intelligible fashion and written in standard English.

Authors: Dear reviewer, we thank you for your constructive comments, suggestion and devoted times. Here, we are happy to respond your comments.

Comments 1: However, there were lots of minor typos. Thoroughly review is needed.

For example, in the abstract Background section 

� Line 7 - change "variations were seen between the studies" to "variations were seen among the studies"

� Line 8 - change incidence of lost follow-up to "incidence of lost to follow-up". Insert to between lost and follow up

Author’s response: Dear reviewer, thank you very much for your constructive comments and correction. Corrections were made in the revised manuscript as per your suggestion.

Comments 2: Method -

� Line 1 - change "we searched" to "We searched" with capital W

� Line 5 - change "Fixed effect models" to "fixed effect models" with a small case letter 'f'

� Similar errors were observed in the abstract. Do correct.

Author’s response: Dear reviewer, thank you very much for your constructive comments and correction. Corrections were made in the revised manuscript as per your suggestion.

Comments 3: Line 69, change 'different' effort to 'several or much or various or multiple' effort

Line 71, change "By" to small case b'' as in by.

Author’s response: Dear reviewer, thank you very much for your constructive comments and correction. Corrections were made in the revised manuscript as per your suggestion, please look on page 4 on line number 68.

Comments: It will be okay to define 'fair or poor ART treatment'

Author’s response: Dear reviewer, thank you very much for your constructive comments and suggestion. Comment were accepted and corrections were made in the revised manuscript as per your suggestion, please look on page 8 from line numbers 152-155.

Attachment Submitted filename: Response to reviewers final.docx

# Peer review: Decision Letter 1

**DOI:** 10.1371/journal.pone.0304239.r003

**Authors:** Zewdu Gashu Dememew

2 May 2024

PONE-D-23-40483R1Incidence of lost to follow up among HIV-positive children on antiretroviral therapy in Ethiopia: systematic review and meta-analysis.PLOS ONE

Dear Dr. Girma,

Thank you for submitting your manuscript to PLOS ONE. After careful consideration, we feel that it has merit but does not fully meet PLOS ONE’s publication criteria as it currently stands. Therefore, we invite you to submit a revised version of the manuscript that addresses the points raised during the review process.

The authors may try to address the following. comments/suggestion before reconsidering the manuscript for publication.

***General Comments and suggestions***

*The author may revise the English language throughout the manuscript: punctuation, grammar… may be rechecked. Revising the manuscript against the journal requirement; specifically, the way reference is listed and written be looked into.*

***Specific comments and suggestions***

*#32, 104, Change ‘November 20/2023’ to November 20, 2023*

*# 42, 104, 116, 144, 168: Change ‘Left to follow up’ and ‘loss to follow-up’ to lost to follow-up and make consistent throughout.*

*#43: why “Counseling’ started with capital letter?*

*#65-67: could be two separate sentences. You may put a full stop after ‘… end of 2022’.*

*# 91-92, 187: check if you need to add ’ after ART initiation’ at end the sentence and the title in # 187. Note there a are a lot of LTFU incidence evidence among HIV children, but fewer while after they put on ART.*

*#122,123: ‘… three 123 authors (GF, ZA, GM, and MS).’ Check if these are these three or four.*

*# 123: you may replace ‘between’ with ‘among’*

*#124: “The each study…’’  what does this mean? Or you may replace ‘The’ with ‘In’*

*#133: ‘Twenty-four studies...’  could be replaced by ‘twenty-four studies...”*

*#159: ‘’…  25, 50, and 75%,’’ make the % uniform to all numbers.*

*#242: check whether “Waster Kenya” is right?*

*#181-288: (limitation): consider revising, summarize this section. May omit numbers 1-5.*

*Reference: Try to revise the write up of the reference per the journal requirement.*

Please submit your revised manuscript by Jun 16 2024 11:59PM. If you will need more time than this to complete your revisions, please reply to this message or contact the journal office at plosone@plos.org. When you're ready to submit your revision, log on to https://www.editorialmanager.com/pone/ and select the 'Submissions Needing Revision' folder to locate your manuscript file.

Please include the following items when submitting your revised manuscript:

- A rebuttal letter that responds to each point raised by the academic editor and reviewer(s). You should upload this letter as a separate file labeled 'Response to Reviewers'.
- A marked-up copy of your manuscript that highlights changes made to the original version. You should upload this as a separate file labeled 'Revised Manuscript with Track Changes'.
- An unmarked version of your revised paper without tracked changes. You should upload this as a separate file labeled 'Manuscript'.

If you would like to make changes to your financial disclosure, please include your updated statement in your cover letter. Guidelines for resubmitting your figure files are available below the reviewer comments at the end of this letter.

If applicable, we recommend that you deposit your laboratory protocols in protocols.io to enhance the reproducibility of your results. Protocols.io assigns your protocol its own identifier (DOI) so that it can be cited independently in the future. For instructions see: https://journals.plos.org/plosone/s/submission-guidelines#loc-laboratory-protocols. Additionally, PLOS ONE offers an option for publishing peer-reviewed Lab Protocol articles, which describe protocols hosted on protocols.io. Read more information on sharing protocols at https://plos.org/protocols?utm_medium=editorial-email&utm_source=authorletters&utm_campaign=protocols.

We look forward to receiving your revised manuscript.

Kind regards,

Zewdu Gashu Dememew, M.D,PhD

Academic Editor

PLOS ONE

Journal Requirements:

Please review your reference list to ensure that it is complete and correct. If you have cited papers that have been retracted, please include the rationale for doing so in the manuscript text, or remove these references and replace them with relevant current references. Any changes to the reference list should be mentioned in the rebuttal letter that accompanies your revised manuscript. If you need to cite a retracted article, indicate the article’s retracted status in the References list and also include a citation and full reference for the retraction notice.

[Note: HTML markup is below. Please do not edit.]

Reviewers' comments:

Reviewer's Responses to Questions

**Comments to the Author**

1. If the authors have adequately addressed your comments raised in a previous round of review and you feel that this manuscript is now acceptable for publication, you may indicate that here to bypass the “Comments to the Author” section, enter your conflict of interest statement in the “Confidential to Editor” section, and submit your "Accept" recommendation.

Reviewer #1: (No Response)

**********

2. Is the manuscript technically sound, and do the data support the conclusions?

The manuscript must describe a technically sound piece of scientific research with data that supports the conclusions. Experiments must have been conducted rigorously, with appropriate controls, replication, and sample sizes. The conclusions must be drawn appropriately based on the data presented. 

Reviewer #1: Partly

**********

3. Has the statistical analysis been performed appropriately and rigorously? 

Reviewer #1: Yes

**********

4. Have the authors made all data underlying the findings in their manuscript fully available?

The PLOS Data policy requires authors to make all data underlying the findings described in their manuscript fully available without restriction, with rare exception (please refer to the Data Availability Statement in the manuscript PDF file). The data should be provided as part of the manuscript or its supporting information, or deposited to a public repository. For example, in addition to summary statistics, the data points behind means, medians and variance measures should be available. If there are restrictions on publicly sharing data—e.g. participant privacy or use of data from a third party—those must be specified.

Reviewer #1: Yes

**********

5. Is the manuscript presented in an intelligible fashion and written in standard English?

PLOS ONE does not copyedit accepted manuscripts, so the language in submitted articles must be clear, correct, and unambiguous. Any typographical or grammatical errors should be corrected at revision, so please note any specific errors here.

Reviewer #1: Yes

**********

6. Review Comments to the Author

Please use the space provided to explain your answers to the questions above. You may also include additional comments for the author, including concerns about dual publication, research ethics, or publication ethics. (Please upload your review as an attachment if it exceeds 20,000 characters)

Reviewer #1: Most of the comments have been addressed. However, the fact that LTFU incidence is low doesn't mean that retention was optimal. This is because the operational definition of LTFU is re-defined as those who interrupted treatment and has not been classified as dead or transferred out. This means, LTFU incidence in this meta-analysis measures cases whose tracing outcome remains unknown. This is a positive finding for the tracing mechanism put in place since deaths, and transfer out cases are being identified and reported. However, the way the paper is written, it seems to suggest LTFU was "generally low" for children with HIV. This is far from reality since retention among children with HIV is far lower that adults with HIV and adult retention at 12 months has not been more than 85% in many cases.

In order to avoid this confusion, clearly state this in the discussion section that

1. LTFU means those with unknown tracing outcome

2. LTFU is not a reflection of improved retention in care rather the improved tracing and reporting mechanism

Finally, make sure to proof read the paper since words like 'ADIS' still remain in the revised version, which should have been edited out as per reviewers comment.

**********

7. PLOS authors have the option to publish the peer review history of their article (what does this mean?). If published, this will include your full peer review and any attached files.

If you choose “no”, your identity will remain anonymous but your review may still be made public.

**Do you want your identity to be public for this peer review?** For information about this choice, including consent withdrawal, please see our Privacy Policy.

Reviewer #1: **Yes: **Kesetebirhan Delele Yirdaw

**********

[NOTE: If reviewer comments were submitted as an attachment file, they will be attached to this email and accessible via the submission site. Please log into your account, locate the manuscript record, and check for the action link "View Attachments". If this link does not appear, there are no attachment files.]

While revising your submission, please upload your figure files to the Preflight Analysis and Conversion Engine (PACE) digital diagnostic tool, https://pacev2.apexcovantage.com/. PACE helps ensure that figures meet PLOS requirements. To use PACE, you must first register as a user. Registration is free. Then, login and navigate to the UPLOAD tab, where you will find detailed instructions on how to use the tool. If you encounter any issues or have any questions when using PACE, please email PLOS at figures@plos.org. Please note that Supporting Information files do not need this step.

Attachment Submitted filename: Incidence of lost to follow up among HIV_min rev.docx

# Author comment: Author response to Decision Letter 1

**DOI:** 10.1371/journal.pone.0304239.r004

3 May 2024

Academic editor comments and respective author’s response

We thank you very much for your big constructs and your constructive comments 

Editor comment 1: The author may revise the English language throughout the manuscript: punctuation, grammar… may be rechecked. Revising the manuscript against the journal requirement; specifically, the way reference is listed and written be looked into.

Authors Response: Thanks very much for this comment. The punctuation, grammar was rigorously rechecked 

Editor comment 2. #32, 104, Change ‘November 20/2023’ to November 20, 2023

Authors Response: dear editors, thank you for your corrective comments. Correction was made in the revised manuscript.

Editors comments: # 42, 104, 116, 144, 168: Change ‘Left to follow up’ and ‘loss to follow-up’ to lost to follow-up and make consistent throughout.

 Authors Response: dear editors, thank you for your corrective comments. Correction was made in the throughout the revised manuscript 

Editor comment 4: #43: why “Counseling’ started with capital letter?.

 Authors Response: dear editors, thank you for your corrective comments. Correction was made in the revised manuscript.

Editor’s comment: #65-67: could be two separate sentences. You may put a full stop after ‘… end of 2022’.

Authors Response: dear editors, thank you for your corrective comments. Correction was made in the revised manuscript.

Editors comments: # 91-92, 187: check if you need to add ’ after ART initiation’ at end the sentence and the title in # 187. Note there a are a lot of LTFU incidence evidence among HIV children, but fewer while after they put on ART.

Authors Response: dear editors, thank you for your corrective comments. Correction was made in the throughout the revised manuscript 

Editor comments : #122,123: ‘… three 123 authors (GF, ZA, GM, and MS).’ Check if these are these three or four.# 123: you may replace ‘between’ with ‘among’

Authors Response: dear editors, thank you for your corrective comments. Correction was made in the revised manuscript.

Editor comments: #124: “The each study…’’ what does this mean? Or you may replace ‘The’ with ‘In’

Authors Response: dear editors, thank you for your corrective comments. Correction was made in the revised manuscript.

Editor comments: #133: ‘Twenty-four studies...’ could be replaced by ‘twenty-four studies...”

Authors Response: dear editors, thank you for your corrective comments. Correction was made in the revised manuscript.

Editors comments: #159: ‘’… 25, 50, and 75%,’’ make the % uniform to all numbers.

Authors Response: dear editors, thank you for your corrective comments. Correction was made in the revised manuscript.

Editor comments: #242: check whether “Waster Kenya” is right?

Authors Response: dear editors, thank you for your corrective comments. Correction was made in the revised manuscript.

Authors Response: dear editors, thank you for your corrective comments. Correction was made in the revised manuscript.

Editor comments : #181-288: (limitation): consider revising, summarize this section. May omit numbers 1-5.

Authors Response: dear editors, thank you for your corrective comments. Correction was made in the revised manuscript.

Editor comments Reference: Try to revise the write up of the reference per the journal requirement.:

Please review your reference list to ensure that it is complete and correct. If you have cited papers that have been retracted, please include the rationale for doing so in the manuscript text, or remove these references and replace them with relevant current references. Any changes to the reference list should be mentioned in the rebuttal letter that accompanies your revised manuscript. If you need to cite a retracted article, indicate the article’s retracted status in the References list and also include a citation and full reference for the retraction notice.

Authors Response: dear editors, thank you for your constructive comments. Dear editors, we didn’t used any retracted and we made revision on the write up of the reference in the revised manuscript. 

Reviewer #1 comments and an author response

We thank you very much for your big constructs and your devoting time to review our manuscript. 

Comment 1: Reviewer #1: Most of the comments have been addressed. However, the fact that LTFU incidence is low doesn't mean that retention was optimal. This is because the operational definition of LTFU is re-defined as those who interrupted treatment and has not been classified as dead or transferred out. This means, LTFU incidence in this meta-analysis measures cases whose tracing outcome remains unknown. This is a positive finding for the tracing mechanism put in place since deaths, and transfer out cases are being identified and reported. However, the way the paper is written, it seems to suggest LTFU was "generally low" for children with HIV. This is far from reality since retention among children with HIV is far lower that adults with HIV and adult retention at 12 months has not been more than 85% in many cases.

In order to avoid this confusion, clearly state this in the discussion section that

1. LTFU means those with unknown tracing outcome

2. LTFU is not a reflection of improved retention in care rather the improved tracing and reporting mechanism

Authors Response: dear reviewer, thank you for your concern, dear reviewer, and correction was made in the revised manuscript, please, look on page 14 from lines numbers 242 to 246

Comment 2: Finally, make sure to proof read the paper since words like 'ADIS' still remain in the revised version, which should have been edited out as per reviewers comment.

Authors Response: dear reviewer, thank you for your corrective comments. Correction was made in the revised manuscript.

Attachment Submitted filename: Response to reviewers final1.docx

# Editor report: Decision Letter 2

**DOI:** 10.1371/journal.pone.0304239.r005

**Authors:** Zewdu Gashu Dememew

9 May 2024

Incidence of lost to follow up among HIV-positive children on antiretroviral therapy in Ethiopia: systematic review and meta-analysis.

PONE-D-23-40483R2

Dear Dr. Author,

We’re pleased to inform you that your manuscript has been judged scientifically suitable for publication and will be formally accepted for publication once it meets all outstanding technical requirements.

Within one week, you’ll receive an e-mail detailing the required amendments. When these have been addressed, you’ll receive a formal acceptance letter and your manuscript will be scheduled for publication.

An invoice will be generated when your article is formally accepted. Please note, if your institution has a publishing partnership with PLOS and your article meets the relevant criteria, all or part of your publication costs will be covered. Please make sure your user information is up-to-date by logging into Editorial Manager at Editorial Manager® and clicking the ‘Update My Information' link at the top of the page. If you have any questions relating to publication charges, please contact our Author Billing department directly at authorbilling@plos.org.

If your institution or institutions have a press office, please notify them about your upcoming paper to help maximize its impact. If they’ll be preparing press materials, please inform our press team as soon as possible -- no later than 48 hours after receiving the formal acceptance. Your manuscript will remain under strict press embargo until 2 pm Eastern Time on the date of publication. For more information, please contact onepress@plos.org.

Kind regards,

Zewdu Gashu Dememew, M.D, PhD

Academic Editor

PLOS ONE

# Editor report: Acceptance letter

**DOI:** 10.1371/journal.pone.0304239.r006

**Authors:** Zewdu Gashu Dememew

10 May 2024

PONE-D-23-40483R2 

PLOS ONE

Dear Dr. Girma, 

I'm pleased to inform you that your manuscript has been deemed suitable for publication in PLOS ONE. Congratulations! Your manuscript is now being handed over to our production team.

At this stage, our production department will prepare your paper for publication. This includes ensuring the following:

* All references, tables, and figures are properly cited

* All relevant supporting information is included in the manuscript submission,

* There are no issues that prevent the paper from being properly typeset

If revisions are needed, the production department will contact you directly to resolve them. If no revisions are needed, you will receive an email when the publication date has been set. At this time, we do not offer pre-publication proofs to authors during production of the accepted work. Please keep in mind that we are working through a large volume of accepted articles, so please give us a few weeks to review your paper and let you know the next and final steps. 

Lastly, if your institution or institutions have a press office, please let them know about your upcoming paper now to help maximize its impact. If they'll be preparing press materials, please inform our press team within the next 48 hours. Your manuscript will remain under strict press embargo until 2 pm Eastern Time on the date of publication. For more information, please contact onepress@plos.org.

If we can help with anything else, please email us at customercare@plos.org.

Thank you for submitting your work to PLOS ONE and supporting open access. 

Kind regards, 

PLOS ONE Editorial Office Staff

on behalf of

Dr. Zewdu Gashu Dememew 

Academic Editor

PLOS ONE
